# DNA origami signposts for identifying proteins on cell membranes by electron cryotomography

**DOI:** 10.1016/j.cell.2021.01.033

**Published:** 2021-02-18

**Authors:** Emma Silvester, Benjamin Vollmer, Vojtěch Pražák, Daven Vasishtan, Emily A. Machala, Catheryne Whittle, Susan Black, Jonathan Bath, Andrew J. Turberfield, Kay Grünewald, Lindsay A. Baker

**Affiliations:** 1 Oxford Particle Imaging Centre, Division of Structural Biology, University of Oxford, Wellcome Centre for Human Genetics, Roosevelt Drive, Oxford UK OX3 7BN; 2 Department of Physics, University of Oxford, Clarendon Laboratory, Parks Road, Oxford UK OX1 3PU; 3 Centre for Structural Systems Biology, Heinrich-Pette-Institut, Leibniz-Institut für Experimentelle Virologie, Notkestrasse 85, 22607 Hamburg, Germany; 4 Department of Chemistry, University of Hamburg, Martin-Luther-King Platz 6, 20146 Hamburg, Germany

**Keywords:** aptamers, cryoEM, cellular electron cryotomography, DNA origami, electron cryomicroscopy, labeling, molecular arrows, protein localisation, signpost origami tags, tagging

## Abstract

Electron cryotomography (cryoET), an electron cryomicroscopy (cryoEM) modality, has changed our understanding of biological function by revealing the native molecular details of membranes, viruses, and cells. However, identification of individual molecules within tomograms from cryoET is challenging because of sample crowding and low signal-to-noise ratios. Here, we present a tagging strategy for cryoET that precisely identifies individual protein complexes in tomograms without relying on metal clusters. Our method makes use of DNA origami to produce “molecular signposts” that target molecules of interest, here via fluorescent fusion proteins, providing a platform generally applicable to biological surfaces. We demonstrate the specificity of signpost origami tags (SPOTs) *in vitro* as well as their suitability for cryoET of membrane vesicles, enveloped viruses, and the exterior of intact mammalian cells.

## Highlights

• Asymmetric DNA signpost origami tags (SPOTs) precisely localize proteins
• SPOTs identify specific proteins in electron cryomicroscopy
• SPOTs have a high contrast “sign” and functionalized “post” base for targeting
• SPOTs recognize fluorescent fusion proteins on vesicles, viruses, and cell surfaces

## Graphical Abstract

**Figure:**
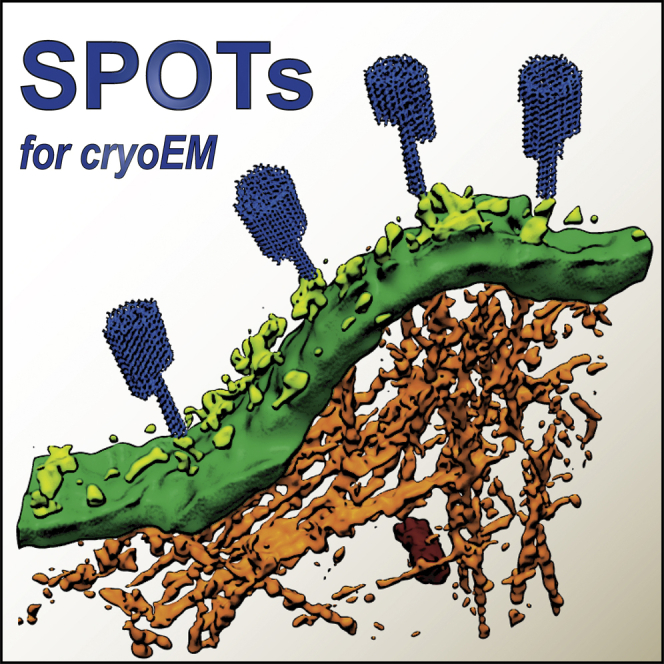

## Teaser

Large, folded DNA structures, designed to recognize proteins on cell surfaces, facilitate *in situ* structural analyses.

## Introduction

Electron cryotomography (cryoET) is a modality of electron cryomicroscopy (cryoEM) where a series of tilted electron microscopic images of a frozen, hydrated specimen are computationally combined into a three-dimensional (3D) volume. Because no averaging is needed for 3D reconstruction, it is especially well-suited to studying pleiomorphic biological structures such as cells, viruses, and membrane vesicles. CryoET is routinely used to reveal molecular details in complex biological systems (Weber et al., 2019), including eukaryotic cellular structures, such as the Golgi (Engel et al., 2015), nuclear pores (von Appen et al., 2015), coat protein complex I (COPI) (Bykov et al., 2017), caveolae (Stoeber et al., 2016), and retromers (Kovtun et al., 2018) and events including trafficking (Grange et al., 2017), translation (Pfeffer et al., 2017), and virus entry (Maurer et al., 2008; Strauss et al., 2015). With sufficient numbers of repeating biological features, sub-volume averaging can be used to improve the resolution of these structures (Schur, 2019), sometimes to sub-nanometer resolution (Bykov et al., 2017; Kovtun et al., 2018; Schur et al., 2015, 2016). The resulting higher-resolution structures can then be plotted back into the original tomogram, providing additional information about molecular organization.

In cryoET, electrons are scattered directly off the native atoms in the biological sample without stains or resin embedding. As a result, every molecule in the sample contributes to the images and reconstructed volume. In crowded biological environments it is often difficult to identify small features of interest or those without a distinct appearance, especially without prior structural information. Correlative methods, such as a combination with cryogenic fluorescence microscopy (cryoCLEM) (Wolff et al., 2016), can be used to identify regions or events of interest. However, technical challenges, including required laser powers and objective lens design, inhibit the widespread use of super-resolution cryoCLEM methods. At best, the localization precision under cryogenic conditions is currently around 50 nm (Schellenberger et al., 2014), insufficient to resolve individual molecules in most cases.

**Figure S1:**
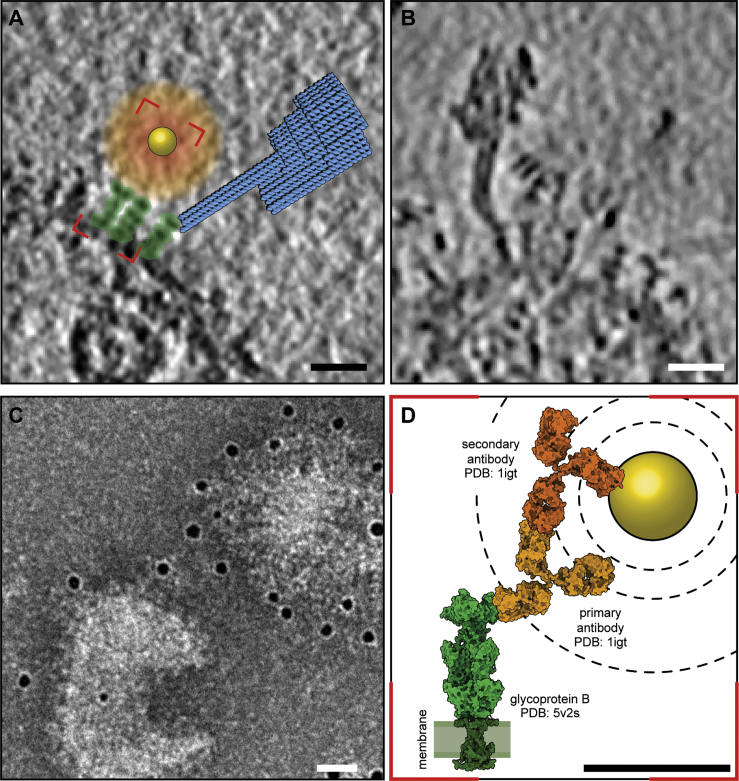
Comparison between immunolabeling and labeling with signpost origami tags (SPOTs), related to Figure 1 (A) Schematic diagram of the two labeling schemes. (B) Labeling of gB vesicles with SPOTs in cryoET, as in Figure 4. The end of the post of the SPOT indicates the position of the labeled protein. (C) Immunolabeling of gB vesicles by negative stain. (D) Surface representation of the crystal structures of the protein being labeled (gB, green) and primary (yellow) and secondary (orange) antibodies in a putative labeling arrangement. Scale bars, 20 nm.

Because of these challenges in identifying features of interest, tagging plays an important role in biological electron microscopy. Several tags have been used with traditional fixed and stained samples, including mini Singlet Oxygen Generator (miniSOG) (Shu et al., 2011), metal-tagging transmission electron microscopy (METTEM) (Risco et al., 2012), metallothionein (Morphew et al., 2015), FerriTag (Clarke and Royle, 2018), and Viper (Doh et al., 2018), but none of these methods are suitable for frozen hydrated samples. A smaller number of tags have been proposed or used for cryoET, including ferritin protein fusions (Wang et al., 2011) and “native” immunogold labeling (Bos et al., 2014; Yi et al., 2015). Ferritin, when genetically fused to the gene of the protein of interest, provides a very high-contrast tag in bacteria, but the required iron concentrations are typically toxic to mammalian cells. The localization precision of traditional immunogold labeling using primary and secondary antibodies is limited to ∼20 nm by the distance between the gold and the antigen recognition site, and the low contrast of the antibodies themselves. This ambiguity generates a large sphere of possible localizations of the molecule of interest in relation to the observed gold nanoparticle (Figure S1). Gold nanoparticles can also be conjugated directly to primary antibodies or antibody fragments (FAbs) (Baschong and Wrigley, 1990; Hainfeld and Furuya, 1992), nanobodies (Goossens et al., 2017; Kijanka et al., 2017), oligonucleotides (Alivisatos et al., 1996; Azubel and Kornberg, 2016; Mirkin et al., 1996), or other small molecules (Azubel et al., 2019; Dahan et al., 2018), which would reduce this distance. Physical adsorption is often used to conjugate colloidal gold to proteins such as antibodies (Oliver, 2010) but is susceptible to dissociation (Park, 1989) because it relies on the hydrophobic effect. Another tag has been proposed that would use glutathione reductase to produce Se-based nanoparticles at the site of interest (Ni et al., 2015), but this has not yet been demonstrated. All of these tagging methods rely on metal atom clusters, which give rise to significantly more scattering than the atoms of the biological sample. Metal clusters can obscure the sample and produce artifacts during tomogram reconstruction (Fernandez et al., 2016) and, because they are roughly spherical, can indicate a general localization rather than a specific position without other contextual information.

Here, we set out to develop a method, suitable for cryoET, to label or tag proteins, enabling their identification on crowded biological surfaces. Critically, we wanted our method to be compatible with frozen hydrated sample preparation and imaging, and not to rely on stains or metals. The tags must be easily identified in projection images and tomograms but not occlude or obstruct the biological structure of interest. Because these tags are to be introduced before vitrification, to be used with cells they would have to be non-toxic with minimal side effects. To be widely applicable, these tags should take advantage of commonly used protein modifications such as green fluorescent protein (GFP) fusions. Most importantly, they must give precise localization with high confidence.

To overcome the limitations associated with toxicity and opacity of existing tags, we decided to investigate tags composed solely of biomolecules. Phosphorus scatters elastically ∼4× more electrons than carbon, oxygen, or nitrogen (National Institutes of Standards and Technology USA, https://srdata.nist.gov/SRD64/Elastic). We reasoned that the phosphate backbone in nucleic acids could provide significant contrast with minimal obstruction of the structures around them, as seen previously in cryoEM of DNA and DNA-protein nanostructures (Bai et al., 2012; Kato et al., 2009; Malo et al., 2005; Martin et al., 2016; Selmi et al., 2011). DNA and RNA can be used to construct elaborate nanostructures (Douglas et al., 2009a; Geary et al., 2014; Han et al., 2017; Ke et al., 2009, 2012; Langecker et al., 2012; Rothemund, 2006; Zhang et al., 2015) by using sequence complementarity to form and link secondary and tertiary structures. Nucleic acid self-assembly therefore has the potential to create biocompatible nanostructure tags with appropriate contrast and distinctive shapes that should be identifiable in a cellular tomogram.

Positions on the surface of nucleic acid nanostructures are uniquely identified through the base sequences of the oligonucleotide components. Functional targeting moieties can thus be located with sub-nanometer precision (Niemeyer, 2010), precisely defining the site of interaction with the target molecule or structure. Control of the stoichiometry of the targeting molecules will also preclude “clustering” effects, which can be induced by multivalent tags such as ferritin (Wang et al., 2011). Aptamers (Ellington and Szostak, 1990; Tuerk and Gold, 1990) provide a potent, adaptable targeting strategy. Aptamers are short nucleic acid sequences that are selected *in vitro* to bind specific molecules. Although the affinities of aptamers for their targets vary widely, published aptamers to standard protein fusion tags (Srisawat and Engelke, 2001; Tan et al., 2012; Tsuji et al., 2009) include, for example, a high-affinity aptamer to standard fluorescent proteins including GFP and yellow fluorescent protein (YFP) (Shui et al., 2012).

**Figure 1:**
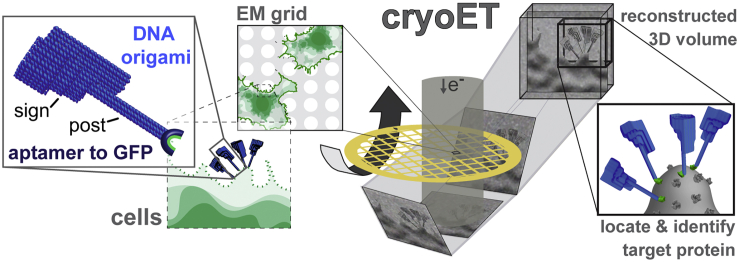
Signpost origami tagging A DNA origami nanostructure, with a “sign” for contrast and identification and a “post” whose base contains an RNA aptamer that binds specifically to a molecular target, is added to cells containing target proteins. The signpost origami tags (SPOTs) are used to identify the proteins of interest in a 3D volume of the sample generated by cryoET.

Here, we describe the development of a nucleic-acid-based tag for cryoET. We have used DNA origami to construct a “signpost” structure, where the “sign” provides the signal for identification in cryoEM images and the bottom of the post is linked to an RNA aptamer that targets common fluorescent proteins (Shui et al., 2012) (Figure 1). We characterize the structure and aptamer-based targeting of our signpost origami tags (SPOTs) and demonstrate their use to tag fluorescent fusion proteins on native membrane vesicles, an enveloped virus and the surfaces of eukaryotic cells.

## Results

### Design and characterization of origami shapes for cryoEM

We designed the signpost tags by using the DNA origami method (Rothemund, 2006), which enables robust assembly of large and complex nanostructures. In this technique, a long “scaffold” strand is folded into a designed shape through hybridization to many complementary “staple” strands. Each staple binds two or more domains on the scaffold, bringing distant regions of the sequence into close proximity. Among many alternative architectures, this technique can be used to construct multilayer nanostructures comprising sets of interconnected parallel helices arranged on a square (Ke et al., 2009) or honeycomb (Douglas et al., 2009a) lattice.

**Figure S2:**
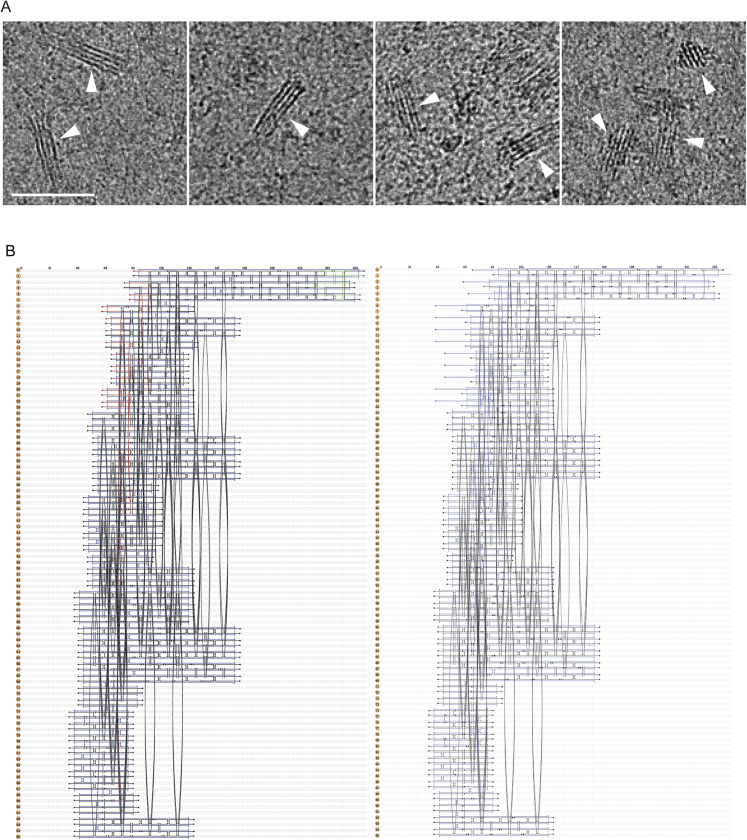
Design of the origami nanostructure, related to Figure 2 (A) CryoEM projection image of the ‘wedge’ origami nanostructure used for initial characterization. The wedge (white arrowheads) was frozen in concentrated cell lysate to determine the contrast of these structures in high-density backgrounds. Scale bar 100 nm. (B) Schematic diagram of staple and scaffold connections for the signpost origami design shown in Figure 2, as generated in caDNAno2 (Douglas et al., 2009b). The first schematic shows the staple layout for the unfunctionalized signpost origami (SPO), the second schematic shows the staple layout for the functionalized signpost origami tag (SPOT) with a Cy5 fluorescent label. The M13mp18 scaffold strand is depicted in light blue. Staples belonging to mixes 1&2, 4, 5, 6 and 7&8 (Table S1) are depicted in black, red, green, dark blue, and pink, respectively.

Nanostructures based on these lattice architectures are dense and rigid. To investigate their suitability as markers for cryoEM, we initially designed and assembled a simple rectangular wedge of ∼90 nm long × 30 nm wide × 20 nm maximum thickness. Because of their periodic structure, the wedges were easily recognized in cryoEM projection images after vitrification in cell lysate (Figure S2A), demonstrating that these lattices are a suitable option for tag design. These observations inspired our subsequent signpost structure, which was designed to maintain these approximate dimensions but incorporate sufficient asymmetry that the orientation of the structure could be uniquely determined in three dimensions. The center of mass of the structure was moved away from the targeting end to allow tagging of closely spaced molecules without spatial conflicts.

**Figure 2:**
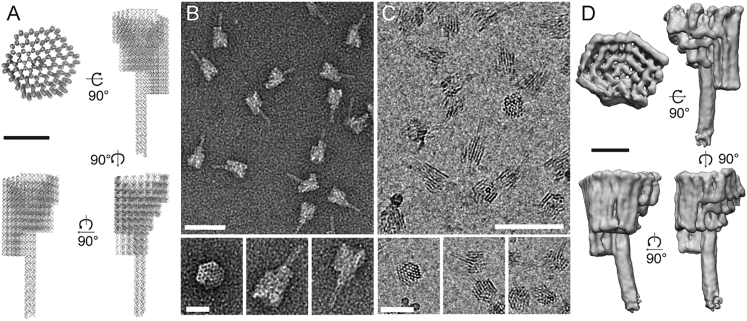
Design and structural characterization of signpost origami (A) Four views of a ribbon (backbone) and plank (base pairs) representation of the signpost origami design. Scale bar, 20 nm. (B) Negative-stain EM projection image (top; scale bar, 100 nm) and selected particles in different orientations (bottom; scale bar, 25 nm) of the folded signpost origami. (C) CryoEM projection image (top; scale bar, 100 nm) and selected particles (bottom; scale bar, 50 nm) show significant contrast of the folded signpost origami in a background of cell lysate. (D) Four views of a 3D structure of the signpost origami obtained from cryoET and sub-volume averaging. This experimentally determined structure closely matches the designed structure in (A). The DNA design of this signpost origami is given in Figure S2. Scale bar, 10 nm.

The signpost is built from a 7,249-nucleotide scaffold strand (single-stranded M13mp18) hybridized to 238 staple oligonucleotides to form a structure of approximately 5 MDa comprising 96 parallel helices arranged in a honeycomb lattice (Figure 2A). Signpost origami nanostructures were folded by thermal annealing and purified by polyethylene glycol (PEG) precipitation followed by molecular weight cut-off filtration (full protocols provided in STAR methods). Five mM Mg^2+^ was maintained in all subsequent preparations to retain proper folding of the nanostructures. The structure of the signpost origami was first verified by negative-stain electron microscopy (Figure 2B). Nanostructures were subsequently imaged in vitrified cell lysate by cryoEM to confirm that the contrast of the structure was sufficient to allow it to be identified in crowded biological environments (Figure 2C). The structure of the signpost origami was confirmed by cryoET and sub-volume averaging (Figure 2D) and closely matches the designed structure.

Signpost origami tags are made by using well-established, robust methodologies, accessible to any laboratory with a thermocycler and commercial oligonucleotide synthesis services, as described in STAR methods. A complete list of the staple sequences needed to produce the signpost origami with its targeting mechanism, described in the following sections, is provided in Tables S1 and S4 (in a format suitable for commercial orders).

### Targeting of signpost origami to structures of interest

**Figure 3:**
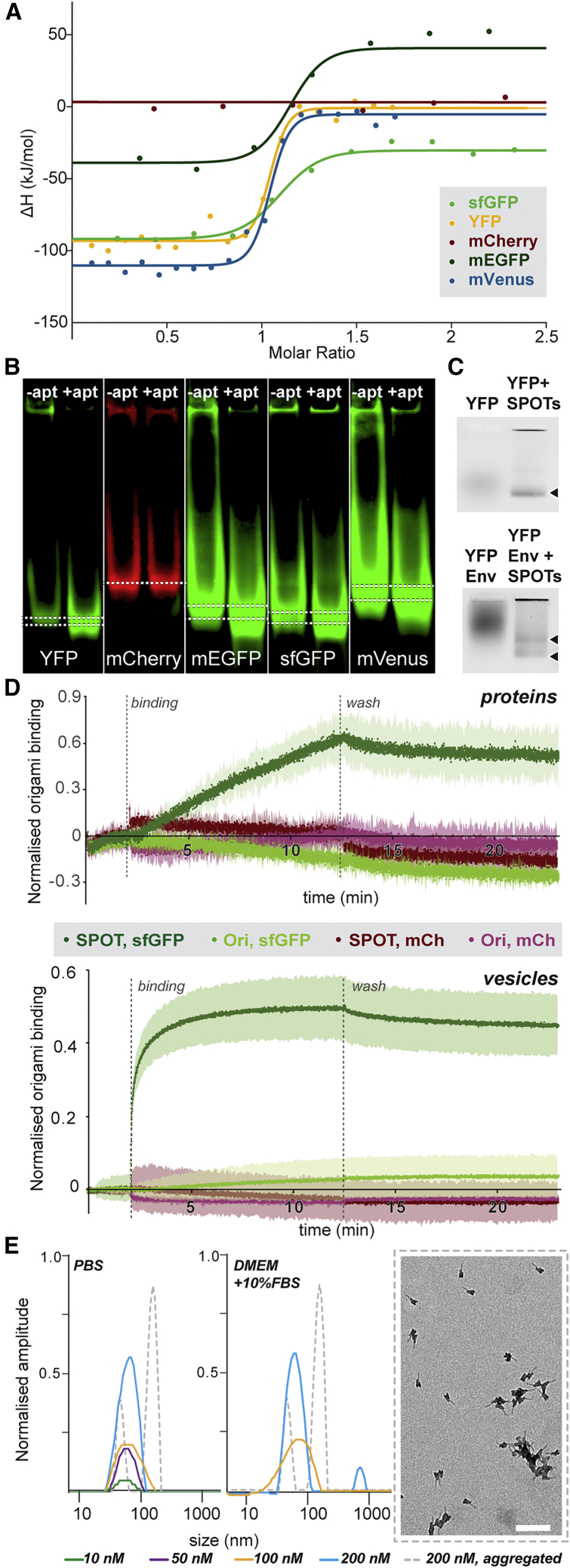
Targeting of fluorescent protein tags by an RNA aptamer The raw data for each experiment can be found in Figure S3. (A) Isothermal titration calorimetry (ITC) of the RNA aptamer alone binding to different fluorescent proteins. (B) Acrylamide gel-shift assay to confirm aptamer binding to fluorescent proteins. The dotted lines indicate bands corresponding to each fluorescent protein without (top line, left column) and with (bottom line, right column) aptamer. (C) Agarose gel-shift assay to confirm binding to fluorescent proteins of the aptamer incorporated into the signpost DNA origami structure. YFP fluorescence was used for detection. Shown at the top is YFP alone; at the bottom is a fusion protein of YFP and Env, the major membrane glycoprotein of murine leukemia virus. YFP and YFP-Env run as sharper bands (black arrow heads) when bound to SPOTs (right lane) than the proteins alone (left lane), as expected from decreased diffusion with SPOTs bound. YFP-Env is expected to run as two species because of the partial isomerization of a disulphide bond, which produces species with both single and triplicate copies of YFP. Some of the DNA nanostructures and associated proteins aggregate in the well (top of image). These samples were run on a single gel; the image is split to better fit figure spacing. (D) Bio-layer interferometry (BLI) was used to confirm the binding of the aptamer-functionalized origami (SPOT) to isolated fluorescent proteins (top) and to a fluorescent protein fusion to glycoprotein B (gB), a surface glycoprotein from herpes simplex virus 1 in native membrane vesicles (bottom). As observed in the other assays, SPOTs bound to the sfGFP or sfGFP-gB much more than signpost origami alone, and SPOTs specifically bound sfGFP or sfGFP-gB, not mCherry or mCherry-gB. Each experiment was run in triplicate, and the lighter region behind each measurement indicates the standard deviation across the three replicates. (E) Dynamic light scattering was used to check for aggregation in concentrated solutions of SPOTs in buffer (left) and cell culture medium (center). A small amount of aggregate was only observed in culture medium at SPOT concentrations of 200 nM, ∼20x higher than needed for cellular experiments (Figure S6). The 50 nM and 10 nM samples were too dilute to observe in culture medium. The aggregation-induced control sample (gray dotted lines) was stained for electron microscopy (right) to monitor the amount of aggregation (scale bar, 200 nm). Such aggregation was not seen in normal preparations of SPOTs (Figures 2B and 2C).

After demonstrating that the designed DNA origami structure was suitable for identification by cryoEM, we investigated how to target these structures to proteins of interest. Because fluorescent proteins are well-studied and commonly used as genetic fusions to proteins of interest, we chose to focus on targeting these proteins to create tools that are broadly applicable to many biological systems. We first tested whether a published RNA aptamer targeting common fluorescent proteins (Shui et al., 2012) could be used. The RNA aptamer was produced by *in vitro* transcription from the corresponding DNA template duplex and purified by ethanol precipitation. Five different fluorescent proteins—super folder GFP (sfGFP) (Pédelacq et al., 2006), monomeric enhanced GFP (mEGFP) (Zacharias et al., 2002), YFP (Ormö et al., 1996), mVenus (Kremers et al., 2006), and mCherry (Shaner et al., 2004)—were produced recombinantly with C-terminal hexahistidine tags and purified from *E. coli* by Ni-nitrilotriacetic acid (Ni-NTA) affinity and size exclusion chromatography. Isothermal titration calorimetry (ITC) was used to characterize binding of the isolated aptamer to each fluorescent protein. Shui et al. (2012) used ITC to measure the binding of this aptamer to the native GFP from the jellyfish *Aequorea victoria* (avGFP). avGFP is dimeric in solution and exhibits different folding behaviors and environmental sensitivity than the fluorescent proteins that subsequently have been engineered as fusion tags. As expected from Shui et al. (2012), the aptamer bound with low nM affinity to mEGFP (k_D_ = 5 ± 6 nM), sfGFP (k_D_ = 13 ± 8 nM), YFP (k_D_ = 4 ± 2 nM), and mVenus (k_D_ = 5 ± 2 nM) (Figure 3A). We saw no evidence of aptamer binding to mCherry, as anticipated from the low sequence conservation between mCherry and the other fluorescent proteins tested. These results were confirmed by native acrylamide gel shift assays, where a change in the mobility of the fluorescent proteins in the presence of the aptamer was observed for sfGFP, mEGFP, YFP, and mVenus but not for mCherry (Figure 3B).

To enable conjugation to the DNA origami nanostructure, a 3′ extension of 12 nucleotides was added to the aptamer sequence. The modified aptamer binds the nanostructure by hybridization of the aptamer extension to a complementary staple extension. Because the molar concentrations needed for ITC were too high to measure binding of intact SPOTs (aptamer-origami conjugates) to fluorescent proteins, we used an agarose gel shift assay (Figure 3C) to confirm that the aptamer was still capable of binding fluorescent proteins after conjugation. The diffuse YFP (detected by YFP fluorescence) sharpens into a band on addition of SPOTs (aptamer-origami conjugates), consistent with binding to the nanostructure tag. A similar effect was observed when the experiment was repeated with detergent-solubilized Env protein, the membrane glycoprotein from murine leukemia virus (MLV), with a surface-exposed YFP tag: this result confirms that binding is not impaired when the fluorescent protein is used as a tag on a target protein. YFP-Env-SPOTs are expected to run as two species due the partial isomerization of a disulphide bond in Env (Pinter and Fleissner, 1977), which produces a mixture of stoichiometrically YFP-tagged trimers (with three SPOTs bound) and monomers (with one SPOT bound).

To confirm target-binding by the aptamer-origami in a more biologically complex setting, we used native membrane vesicles displaying either sfGFP- or mCherry-tagged versions of glycoprotein B (gB), a principal component of the herpes simplex virus 1 (HSV-1) membrane fusion machinery. Vesicles displaying membrane-embedded target proteins were produced by using the MPEEV (membrane protein-enriched extracellular vesicles) system (Zeev-Ben-Mordehai et al., 2014a), exploiting endogenous extracellular vesicle production during membrane protein overexpression. We have used these vesicles previously for structural characterization of HSV-1 gB (Vollmer et al., 2020; Zeev-Ben-Mordehai et al., 2016) and *C. elegans* cell-cell fusion protein EFF-1 (Zeev-Ben-Mordehai et al., 2014b). We created tagged versions of gB with sfGFP or mCherry inserted after the 30-residue N-terminal signal sequence. Upon transfection into HEK293T or BHK-21 cell lines, sfGFP/mCherry-gB vesicles were produced with the fluorescent protein located on the ectodomain of gB, facing the extracellular space. Mass spectrometry has been used previously to show that gB is the only transmembrane protein found at detectable amounts in these vesicles; other soluble proteins are present in the background of the purification as well as inside the vesicles (Zeev-Ben-Mordehai et al., 2014a). We measured binding of SPOTs to purified sfGFP and mCherry or, in context, with gB vesicles by using bio-layer interferometry (BLI) (Figure 3D). As expected, no binding to mCherry was observed either in isolation or as a fusion protein, with or without the aptamer. In comparison with the signpost origami without aptamer, significantly more SPOT binding was observed to sfGFP in isolation and on the gB membrane vesicles. Further, when we dilute the sfGFP-gB vesicles with mCherry-gB vesicles, binding decreases in correspondence with the sfGFP concentration, indicating that SPOTs will also bind to sfGFP when sparsely distributed across a surface (Figure S6A). Altogether, these results suggest that our SPOTs can target GFP and related fluorescent proteins with high affinity and specificity.

**Figure S3:**
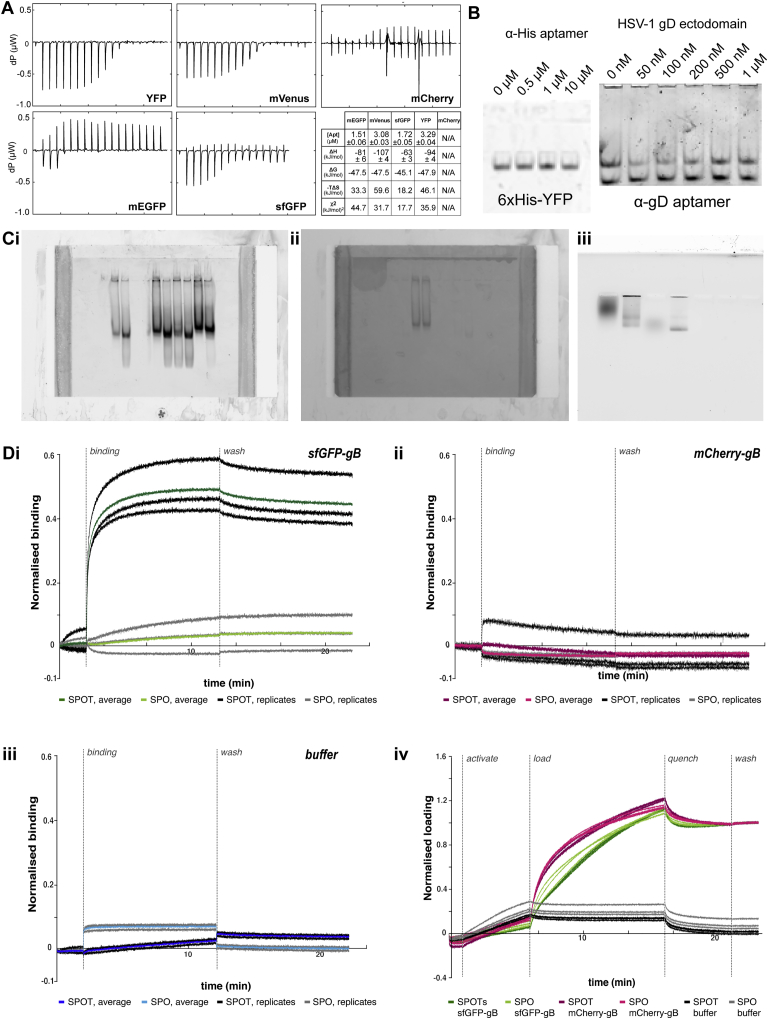
Biophysical data, related to Figure 3 (A) Raw isothermal titration calorimetry data for fluorescent proteins titrated into aptamer (not linked to origami signpost). The fitted parameters are shown in the table for each binding curve. (B) We were unable to confirm binding of other aptamers using acrylamide gel shift assays. Left, an aptamer reported against hexahistidine tags (Tsuji et al., 2009); Right, a reported aptamer against glycoprotein D from Herpes simplex virus I (Yadavalli et al., 2017) Ci-iii. Unprocessed gel images with fluorescent detection in the GFP (i), RFP (ii), and YFP (iii) channels for gel-shift assays in Figures 3B and 3C. iii Lane 3 - YFP alone. Because of its low molecular weight (∼26 kDa), we expect YFP to diffuse in the agarose gel, resulting in a band that thus appears faint and with a lower apparent motility. Lane 4 - upon addition of SPOTs to YFP, the molecular weight of the fluorescent structure would increase dramatically when they bind (∼5 MDa) so it diffuses much less, and a sharper and therefore brighter band would be seen. Lane 1 - YFP-Env has a much higher molecular weight than YFP (∼125 kDa monomer, ∼375 kDa trimer). As both forms of YFP-Env are much larger than YFP, the molecules should diffuse less in the agarose and thus appear as a more concentrated band. Additionally, there are 3 YFP on each Env trimer such that we would expect 3x the signal of YFP alone. Lane 2 - upon addition of SPOTs to YFP-Env, the trimer population of YFP-Env could bind 3 SPOTs (∼15 MDa) and the monomer population could bind one SPOT (∼5 MDa). As YFP-Env is a membrane protein produced in mammalian cells, it is glycosylated and detergent-solubilized here, both of which may impact the appearance of bands by electrophoresis. D. Example replicate buffer-subtracted and loading-normalized traces for BLI binding of unfunctionalized sign post origami (‘SPO’) or SPOTs to immobilized sfGFP-gB vesicles (i) or mCherry-gB vesicles (ii) from Figure 3D (bottom panel). The buffer replicates (unloaded tips dipped in analyte nanostructure solutions) and average (used for subtraction) are show in iii, and the loading curves (normalized in the same way as the data) in *iv*.

**Figure S4:**
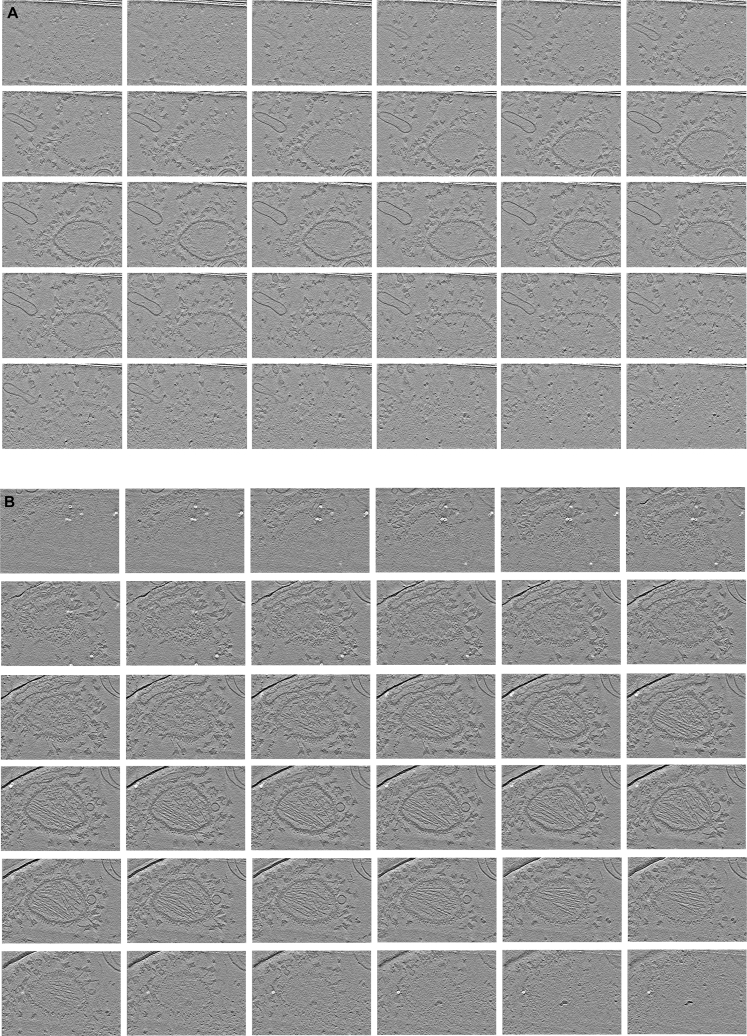
Tomograms of SPOTs binding sfGFP-gB vesicles from Figure 4, shown as a series of computed z-slices with each slice 2.7 nm thick (A) A tomogram from Figure 4D. (B) The tomogram in Figure 4A and B. Gold beads used as fiducial markers have been computationally removed and appear as white spots in some slices due to the contrast enhancement applied for presentation. Field of view in each slice is ∼557 × 783 nm.

**Figure S5:**
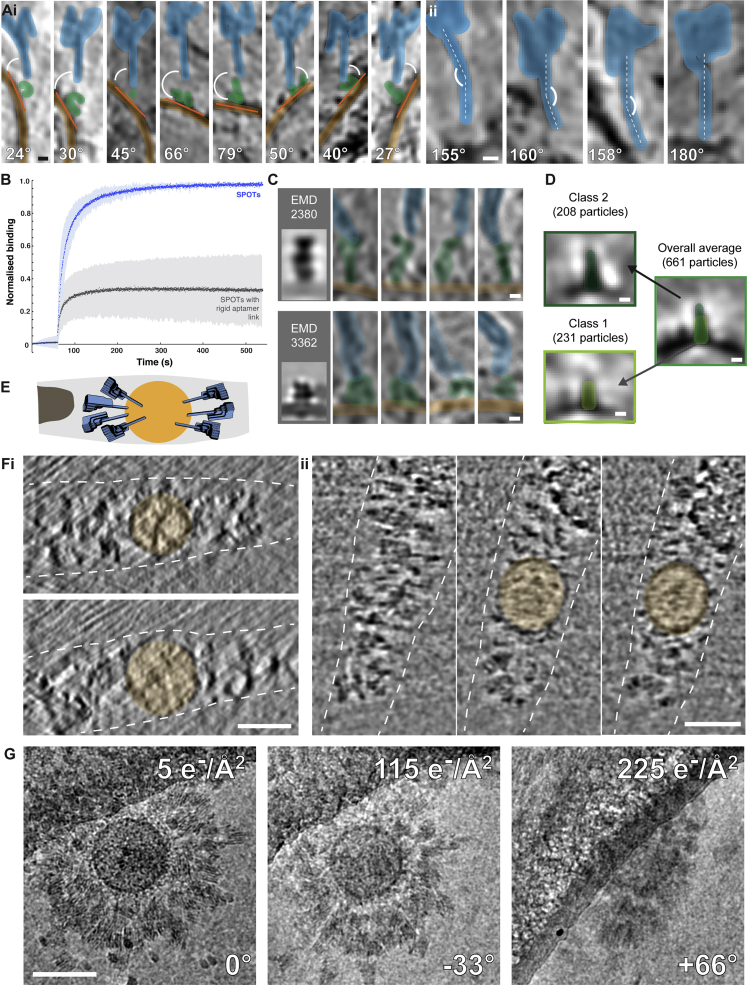
Behavior of SPOTs in cryoET, related to Figures 4 and 5 (A) The flexibility in the link to the aptamer (i) is more pronounced than flexibility in the SPOTs themselves (ii). The variation in the SPOT orientation relative to the membrane was approximately ± 65°, while the bend in the SPOT post was approximately ± 25°. Scale bars 10 nm; computed tomographic slice thickness 9.8 nm. (B) Rigidity in the attachment of the aptamer to the rest of the origami decreases SPOT binding to sfGFP-gB vesicles as measured by BLI. In addition to the original SPOT design, a second design was tested where the aptamer was linked at the 5′ and 3′ ends to the signpost structure. This design (blue line) bound much less to sfGFP-gB vesicles than the flexible SPOT design used throughout (green line). The experiments were repeated in triplicate, and the shaded area behind each curve represents the standard deviation of the three measurements. (C) sfGFP-gB exhibits multiple conformations on vesicles, and SPOTs bind at least two previously identified conformations – top row, a tall conformation (Maurer et al., 2013) (EMD: 2380); bottom row, a short conformation (Zeev-Ben-Mordehai et al., 2016) (EMD: 3362). Scale bars 5 nm, computed slice thickness 2.8 nm. (D) When using SPOTs to estimate the locations of gB, and subjecting these particles to sub-volume averaging, it is possible to obtain two classes (comprising ∼2/3 of the data) that broadly resemble two known conformations of gB, with the overall average a mixture of conformations. Scale bars 5 nm; computed slice thicknesses ∼2 nm. (E) Schematic diagram showing the position of murine leukemia virus (orange) in the ice layer (gray) after vitrification with SPOTs (blue) (tomogram shown in Figure 5). The edge of the hole in the carbon support layer is shown in brown on the left. (F) Example XZ (i) and YZ (ii) slices of the tomogram in Figure 5. The position of the virus is shown in orange and the approximate boundaries of the vitreous ice layer are drawn with dashed white lines. Scale bars 100 nm. (G) Example projection images from a dose-symmetric tilt scheme with high electron exposures, monitoring changes in appearance of SPOTs at high exposure and tilt. After total exposures of > 100 e^-^/Å^2^ (300 kV), the stripe pattern of the DNA helices and the overall shape of the SPOTs are still clearly visible. Scale bar, 100 nm.

**Figure S6:**
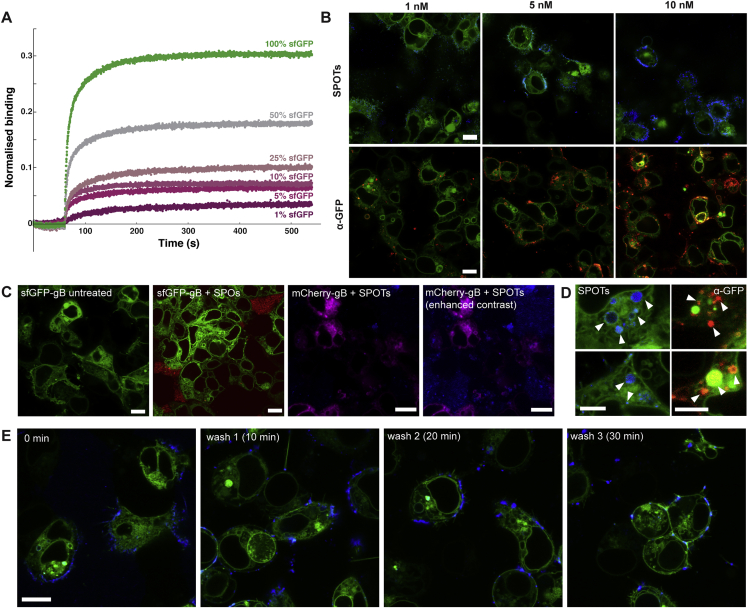
Control experiments for SPOT binding, related to Figure 6 (A) Bio-layer interferometry (BLI) was used to measure the binding of SPOTs as a function of tagged protein concentration. sfGFP-gB vesicles (Figures 3 and 4) were diluted with vesicles with mCherry-gB. Even at 1% sfGFP, a small amount of binding was still detected. (B) Confocal slices from live cell fluorescence microscopy of titrations of HEK293T cells expressing sfGFP-gB (green) with increasing concentrations of Texas Red-labeled SPOTs (blue, top row) and an α-GFP antibody conjugated to Alexa-647 (red, bottom row). Similar patterns of binding were observed for the SPOTs and antibody, reaching maximum binding at ∼10 nM. Scale bars 10 μm. (C) Control experiments for fluorescent SPOT binding to cells. First panel from left – some cells expressing sfGFP-gB (green) exhibit unusual morphologies as a result of gB expression, independent of SPOT or antibody addition. Second panel – when the Texas Red-labeled signpost origami structure is produced without aptamer (SPO, red), it is found in solution and no binding is observed to sfGFP-gB expressing cells (green). The black spaces are occupied by untransfected cells which also occlude the SPO. Third panel – No Cy5-labeled SPOT (blue) binding is observed on cells expressiong mCherry-gB (magenta). The free SPOTs can be seen in the background when the contrast is enhanced (fourth panel). Scale bars 10 μm. (D) Internal cell vesicles (green) were often observed with both Texas Red-labeled SPOT (blue, left) and α-GFP antibody (red, right) signal after incubation. gB undergoes a recycling process from the plasma membrane that appears to not be impeded by SPOTs or the antibody. Scale bars 5 μm. (E) Texas Red-labeled SPOTs (blue) remained bound to sfGFP-gB expressing cells (green) even after the culture medium was fully replaced three times over 30 min. The fresh medium was left for 10 min between washes to allow for dissociation. This continued binding is consistent with the slow dissociation rates measured by BLI for sfGFP-gB vesicles (Figure 3D). Scale bars 10 μm.

Finally, to confirm that SPOTs remained stably folded and did not aggregate under the conditions used for imaging, we used dynamic light scattering (DLS) to monitor their size distribution under different conditions (Figure 3E). No evidence of aggregation was observed in the conditions used for imaging, although a small amount of aggregation was observed in cell culture medium supplemented with fetal bovine serum at SPOT concentrations ∼20× higher than needed for cellular imaging.

### Applications to biological systems I: membrane vesicles

**Figure 4:**
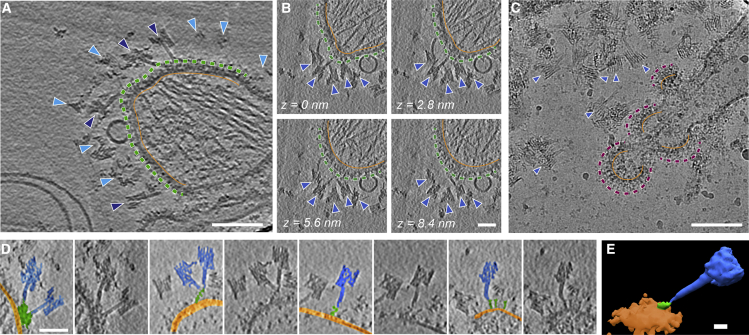
SPOTs binding to sfGFP-gB vesicles observed by cryoET Computed tomogram slices in all panels are 2.8 nm thick. (A) Computed slice from a high-magnification tomogram (1.34 Å/pix) showing SPOTs binding selectively to sfGFP-gB vesicles. The distal edge of sfGFP-gB is indicated by a green dashed line; an orange line is drawn just below the membrane surface. SPOTs are indicated with dark blue arrow heads if their posts are visible in the tomogram slice and light blue if they are situated across multiple slices. Full views of all slices from the tomogram are shown in Figure S4. Average defocus across the tilt series was ∼−3.8 μm. Scale bar, 100 nm. (B) Sequential computed tomogram slices showing SPOTs bound to sfGFP-gB. Structures are labeled as in (A). Scale bar, 50 nm. (C) SPOTs are not observed binding to the vesicles in a projection image when gB is tagged with mCherry instead of sfGFP. Magenta dashed lines indicate the distal protein face, and orange lines indicate the inside of the membrane. mCherry belongs to a different family of fluorescent proteins than sfGFP. Scale bar, 100 nm. (D) Gallery of images of individual SPOTs extracted from tomograms of labeled sfGFP-gB vesicles. Each image is shown twice, with and without annotation on the left and right, respectively. SPOTs are highlighted in blue, sfGFP-gB in green, and the membrane in orange. It is often possible to follow the SPOT posts directly to the recognized protein density on the vesicle surfaces. Scale bar, 50 nm. (E) A sub-volume average of SPOTs (blue) bound to sfGFP-gB (green) in vesicle membrane (orange) with the orientations determined by the positions of the posts of the SPOTs as initial alignment markers. Scale bar, 10 nm.

As a preliminary test of the SPOT tagging method in cryoET, we used the same sfGFP-gB vesicles described above. Vesicles were incubated with SPOTs for 15–30 min on ice and plunge frozen for cryoET. On these vesicles, the target protein gB can be recognized by eye in reconstructed tomograms (Figure 4A). As intended, we frequently observed SPOTs bound to sfGFP-gB on the vesicle surfaces (Figures 4A, 4B, and 4E). When the experiment was repeated with control mCherry-gB, no association between SPOTS and the vesicle membrane was observed despite an abundance of SPOTs (Figure 4C).

SPOTs adopted an array of orientations in relation to the membrane, often spanning multiple z-slices of the tomogram. This variability is most likely due to the flexibility of the N-terminal region of gB where sfGFP is inserted, as well as inherent flexibility in the aptamer and its oligonucleotide linker to the origami (Figure S5Ai). A slight curve was observed in some posts of the SPOTs (Figure S5Aii), but this variability was much smaller (∼25°) than for the link between the targeting and identification portion of the SPOTs (∼65°). To try to reduce the variability in binding angle in relation to the membrane, we designed a “rigid SPOT” in which the aptamer is linked to the signpost at the 5′ and 3′ ends of the aptamer sequence, instead of the single link at one end that was used in the original design. BLI was used to compare the binding to sfGFP-gB vesicles of these rigid SPOTs and the original design (Figure S5B). The rigid SPOTs bound significantly less (∼30% of the original SPOT binding), strongly suggesting that the flexibility of the aptamer and its attachment is important for efficient targeting of proteins of interest on crowded surfaces.

Because no attempt was made to remove SPOTs during sample preparation for cryoET, a small proportion of unbound SPOTs were also observed in the sfGFP-gB sample, either free in solution (< 5%) or interacting with the air-water interface or carbon film, but these were easily identified visually and excluded. The proportion of unbound SPOTs depends on their concentration relative to that of the protein of interest, which is difficult to measure accurately for membrane vesicles. Although some SPOTs were observed binding to the top or bottom surface of the vesicles, the majority (> 90%) were detected on the sides of the vesicles, suggesting that the air-water interface, sample thickness, or blotting might affect binding.

In many cases, the posts of the SPOTs could be directly traced to protein density on the vesicle surface (see Figures 4D and S5C for examples). We therefore decided to evaluate the use of SPOTs in the context of sub-volume averaging. Particles were picked manually at the apparent joint between the base of the SPOT post and the protein on the vesicle surface, and a second point further up the post of each SPOT was used for initial orientation determination. The resultant average showed the SPOTs, membrane, and protein (Figure 4D), but the protein structure was of insufficient quality to identify it conclusively as gB. Using a mask that only included the protein and a small amount of membrane did not produce a coherent average when the SPOTs were used to provide initial orientations. However, if the SPOTs were only used to define the proteins of interest, and their initial orientations were given by a line normal to the membrane (as would be done if the SPOTs were not present), an average was produced that revealed both membrane and proteins (Figure S5D). Glycoprotein B exists in multiple conformations on the membranes (Figure S5C) (Maurer et al., 2013; Zeev-Ben-Mordehai et al., 2016). After several iterations of alignment, classification produced averages with features resembling the two known conformations (Figure S5D), both of which contribute to the overall average. These results suggest that although SPOTs cannot be used as additional rigid density to improve alignment in sub-volume averaging, their presence does not interfere with sub-volume averaging. Further, because most particles for sub-volume averaging are still picked manually, SPOTs might reduce the bias of the experimenter in particle picking, improving the quality of the picked dataset.

### Applications to biological systems II: viruses

**Figure 5:**
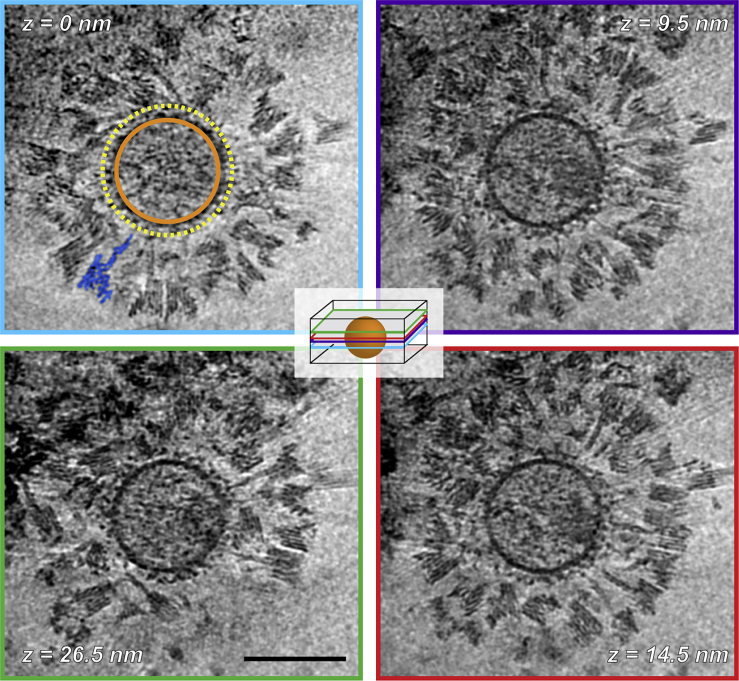
SPOTs binding to YFP-tagged Env in the envelope of murine leukemia virus (MLV) Computed slices through the tomogram reconstructed from the tilt series shown in Figure S5G. The slice position in the reconstructed volume increases in a clockwise manner and is recorded in each panel as text and by the colored frame indicated in the central schematic diagram, where the virus is represented by the orange sphere. In the first panel (z = 0 nm), the inside of the viral membrane and approximate distal surface of the viral glycoproteins tagged with YFP are indicated by the solid orange and dashed yellow circles, respectively. A single SPOT is highlighted in blue. Tomogram slices are ∼0.5 nm thick. Scale bar, 100 nm.

To assess the suitability of SPOTs for targeting proteins on viral surfaces, we used murine leukemia virus (MLV). MLV has a single glycoprotein, Envelope (Env), on its surface, providing a simple test system. To enable SPOT targeting, we used Env displaying YFP inserted into a large extra-viral loop. We incubated recombinant MLV and SPOTs for 30 min before vitrification and imaging by cryoET (Figure 5).

To investigate the radiation stability of SPOTs, we collected dose-symmetric tilt series with high electron exposures (∼225 e^−^/Å^2^ total). Halfway through the tilt series (at >100 e^−^/Å^2^ total exposure), contrast is reduced by radiation damage, but the characteristic stripe pattern of the parallel DNA helices of the SPOTs is still visible. At the highest tilts (>60°, >200 e^−^/Å^2^), the SPOTs are still clearly visible, though the stripes are harder to resolve (Figure S5G).

The resulting tomograms show a strikingly high level of binding by SPOTs. In many slices, the virus surface appeared to be fully covered (Figure 5). SPOT binding does not appear to disrupt the native organization in areas where Env is densely packed; rather, in such regions SPOT binding is occluded. As with the vesicle preparations, most SPOTs were found around the sides of the virus rather than at the top or bottom, most likely again due to blotting or the air-water interface (Figures S5E and S5F). As with the vesicles, many SPOTs can be traced directly to individual proteins on the viral surface, even when they span multiple z slices, confirming that YFP targeting behaves very similarly to sfGFP targeting, as suggested by our biophysical data.

### Applications to biological systems III: mammalian cells

**Figure 6:**
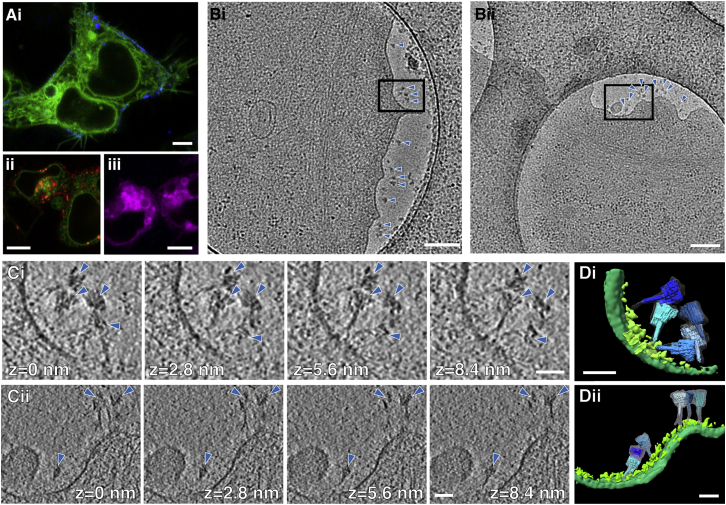
SPOTs binding to cells expressing sfGFP-gB (A) In (i) is a confocal slice from live-cell fluorescence microscopy of HEK293T cells expressing sfGFP-tagged gB from HSV-1 (green) ∼15 min after addition of Cy5-labeled SPOTs (blue). SPOTs quickly localize to the surface of cells expressing sfGFP-gB. In (ii), the pattern of SPOT binding is similar to that observed with α-GFP antibodies conjugated to Alexa-647 (red). In (iii), no evidence of cell surface binding is observed when SPOTs are added to cells expressing mCherry-gB (magenta). Scale bars, 5 μm. (B) Two low-magnification cryoEM projection images of SPOTS bound to the surface of BHK-21 cells expressing sfGFP-gB (i and ii). SPOTs are indicated with blue arrow heads. Scale bar, 250 nm. (C) Sequential computed tomographic slices of the areas indicated in (Bi) and (Bii), respectively. Although the SPOTs (blue arrow heads) often extend across multiple slices, it is still possible to trace the post structure to the membrane in many cases. Slice thickness is ∼2.8 nm. Scale bars, 50 nm. Average defocus across the tilt series is ∼−3.8 and −4.5 μm for (i) and (ii), respectively. (D) Manual segmentation of the tomograms in (C). The signpost origami structure (various shades of blue) from Figure 2 is fit into each of the segmented SPOTs (gray mesh). The plasma membrane is shown in green, with protein density on the membrane surface in light green. Scale bars, 50 nm.

Finally, we investigated the use of SPOTs for cellular cryoET. We transiently transfected HEK293T or BHK-21 cells with the gene for sfGFP-gB used in the vesicle system above. We used confocal fluorescence microscopy to observe co-localization between SPOTs, labeled with the fluorophore Cy5 or Texas Red on an oligonucleotide that hybridizes with 12 staples used to fold the signpost structure (Tables S1 and S2), and the sfGFP-gB on the cell surface (Figure 6Ai). The SPOT signal localized to the cell surface and became especially pronounced in live imaging ∼15 min after addition to cells. This binding was similar to that of an anti-GFP antibody (Figure 6Aii). We performed a titration (Figure S6B) to explore the amount of surface binding that could be achieved with SPOTs and compared it with a titration of an anti-GFP antibody labeled with Alexa-647. Similar patterns of binding were observed for the SPOTs and antibody, which saturated at concentrations of ∼10 nM. Additional control experiments showing sfGFP-gB-expressing cells before SPOT addition and the slow dissociation of SPOTs upon a change in medium are presented in Figure S6. Negative controls using the signpost origami without the aptamer and cells expressing mCherry-gB (Figure S6C) confirmed the binding specificity of SPOTs. The gB protein carries a cytoplasmic endocytosis motif, causing it to be trafficked from the plasma membrane back into the cell via the endocytic pathway (Niazy et al., 2017). At later time points, vesicles inside the cell also displayed SPOT or anti-GFP antibody fluorescence, suggesting that they are endocytosed along with their protein target without affecting this function of gB (Figure S6D).

We next grew BHK-21 cells on EM grids and, after transient transfection with sfGFP-gB, incubated them with SPOTs for ∼15 min and plunge froze. The thin periphery of the cells was then used for cryoET without thinning (Figure 6B). With careful observation at low magnification, SPOTs could be seen bound to the cell surface and these areas were selected for tilt series collection. After tomogram reconstruction, SPOTs were easily observed bound to the plasma membrane. On most cells, SPOTs were not bound uniformly across the cell surface but in small clusters. Some SPOT posts could be traced to protein density on the surface. This protein density would have been difficult to resolve otherwise, more so than with the vesicle and virus imaging described above, due to the increased thickness of the cellular tomograms and corresponding decrease in signal-to-noise ratio.

## Discussion

### Feasibility of DNA origami as a marker for cryoET

The results show that our purpose-designed DNA origami signpost tags can be clearly identified in tomograms of crowded biological surfaces. In projection images, SPOTs are easily identified by the contrast of the repeating stripe patterns generated by the overlap of aligned DNA helices (Figure 2). In tomograms, details to the level of individual DNA helices were visible and the overall shape of the SPOTs was clearly recognizable, and there was sufficient contrast to orient the sub-volume averaging structure by eye. Partially folded or dis-assembled SPOTs were not observed. SPOTs have higher contrast than the surrounding protein and often lipids as a result of the phosphorus in the aligned DNA backbones. However, we did not observe any artifacts in tomogram reconstruction caused by strong scattering that violates the weak phase or amplitude approximations, which can be a problem with gold or other metal clusters (Fernandez et al., 2016). Because of the direct attachment to the target molecule, SPOTs enable precise target localization whereas immunogold labeling puts the gold clusters up to 20 nm away (Figure S1).

### Effectiveness of the aptamer-based targeting of SPOTs

A successful tag enables precise localization with high specificity for its target. For all three of the biological systems tested, the posts of a substantial fraction of bound SPOTs can be traced in 3D to a single protein on a membrane surface. In the absence of the fluorescent protein fusion recognized by the SPOTs, no such binding was observed by cryoEM or other biophysical methods (Figures 3, 4, 6, and S6), confirming the effectiveness of the aptamer-based targeting system. Altogether, these results demonstrate that SPOT targeting is specific and precise.

The aptamer used in our SPOTs successfully binds several popular fluorescent proteins widely used as genetic fusions, including sfGFP, YFP, and mVenus. Notably, it does not interact with other commonly used fluorescent proteins such as mCherry (and by extension, most likely other fluorescent proteins in the “mFruit” series, such as mApple or mGrape) that are not closely related to GFP. This specificity will be particularly advantageous for cryoCLEM applications, enabling the use of multiple fluorescent protein fusions for co-localization experiments without interfering with SPOTs. In cases where events are particularly rare, cryogenic fluorescence microscopy could be used, prior to cryoEM, to identify subcellular locations where fluorescent SPOTs are bound. In general, our results indicate that SPOTs can be used for cellular tagging without the need for cryoCLEM when expression amounts and event numbers are sufficiently high.

### Suitability of SPOTs for tagging different biological systems

Based on the range of biological systems tested here, we expect SPOTs to be suitable for most sample types studied by cryoET. Importantly, we did not observe any toxicity effects when using SPOTs with cells. Each sample required some optimization but, once conditions for freezing had been determined, SPOTs could be added without changing those conditions. We did not find it necessary to wash off unbound SPOTs because they were rare and easy to identify. If necessary, the number of unbound SPOTs could be minimized in cellular samples by replacing the culture medium immediately prior to plunge freezing, given that SPOTs were still observed bound to the cell surface after 30 min and 3 washes (Figure S6E). For vesicles or viruses, a dedicated separation protocol, perhaps involving differential centrifugation, could be implemented if needed. However, because of the equilibrium nature of aptamer binding, a small proportion of unbound SPOTs will always be present in an equilibrated system.

The many biological questions that could be addressed by using SPOTs include protein (co)localization, *de novo* structure detection, identification of isolated sub-cellular membranes, and determination of vesicle or synthetic membrane bilayer orientation. Indeed, SPOTs will be useful in most situations where immunolabeling could be applied, as long as the targeted protein is tractable to genetic tagging. Although untested here, SPOTs could potentially be used to confirm the presence of a subunit in a protein complex in solution, for example before single-particle cryoEM of samples sensitive to disassembly by the air-water interface. This approach would provide an advantage over antibody- or chemically based gold tags because the bulk of the tag is displaced from the subunit of interest and complexes would not have to be reconstituted *in vitro* (e.g., Hölzl et al., 2000). As demonstrated here, SPOTs can be applied in crowded biological environments such as cell surfaces. As SPOTs interact with their targets post-translationally, immediately before observation, they should have little effect on native protein localization beyond that of the original fluorescent protein fusion. In the MLV example studied here (Figure 5), SPOT binding does not disrupt the native organization of densely packed proteins of interest. In cases where SPOT binding is occluded by protein packing, proteins might have a variable number of SPOTs bound, depending on the oligomer number and organization.

### Sub-volume averaging and SPOTs

We have demonstrated here that the SPOTs themselves are suitable for sub-volume averaging (Figure 2). However, we also determined that a flexible link to the aptamer is necessary to maintain optimal binding (Figure S5B). This flexibility, in combination with the linker connecting the fluorescent tag to the protein of interest that is often necessary to maintain biological function, limits the potential for sub-volume averaging of SPOT-target protein complexes. Nevertheless, we have demonstrated here that SPOTs do not interfere with sub-volume averaging of the target protein itself (Figure S5D) and still allow classification. Indeed, there could be cases where sub-volume averaging can be improved by using SPOTs to remove user bias in particle picking. Further, if we had been unaware of the multiple conformations of gB, the SPOTs would have revealed these additional forms of the protein, given that they can easily be seen bound to individual particles of clearly different shapes (Figure S5C). In the future, SPOTs could potentially provide a larger signal for template matching than the protein of interest, allowing for automation and a further reduction in picking bias.

### Potential applications and directions for further development

Looking forward, there are several obvious extensions to the tagging method described here. For example, SPOTs could also be used with stained, embedded cellular samples: DNA stains readily with uranyl acetate (Erenpreisa, 1981), and SPOTs could provide significantly higher localization precision than immunogold labeling. The DNA origami design can be readily adapted to produce tags with different shapes and sizes which, with independent targeting strategies, could be used to identify protein co-localization or otherwise target multiple proteins in a single experiment. Although the aptamer to GFP binds with high affinity, we have been less successful in replicating this behavior with other aptamers we tested (Figure S3B). We hope that this study will encourage development of more robust aptamers to common protein fusion tags. Targeting methods other than aptamers might be developed in the future, giving even broader applications for SPOTs.

An exciting further use of SPOTs would be for intracellular identification of proteins of interest. Because SPOTs are composed entirely of biomolecules themselves, and other DNA nanostructures have successfully been introduced into mammalian cells previously (Jiang et al., 2012; Modi et al., 2009; Schüller et al., 2011; Walsh et al., 2011), biocompatibility and toxicity should not present major hurdles that would preclude using SPOTs inside mammalian cells. Further, our experiments imaging SPOTs in cell lysate (Figure 2C) suggest that the current origami design provides sufficient contrast for cryoEM in a background of cytoplasm. However, the architecture of mammalian cells poses two main challenges for making intracellular use widely applicable. Methodology is needed to allow enough SPOTs to enter the cell for sufficient labeling while not disrupting biological activity. Further, robust delivery of SPOTs to their target organelle or subcellular region is needed. Given that high-yield delivery of diverse molecules into cells is an active area of research, we anticipate future developments will allow SPOT use inside mammalian cells.

In conclusion, SPOTs provide a previously lacking tool for the identification and precise localization of structures of interest in the rapidly developing field of cryoEM. The ability to label and localize molecules with high specificity and precision within a native, hydrated specimen has the potential to greatly expand applications of cryoET. SPOTs also have the potential for use in other branches of biological electron microscopy, including imaging of plastic sections and single-particle cryoEM.

## STAR★Methods

### Key Resources Table

**Table:** 

REAGENT or RESOURCE	SOURCE	IDENTIFIER
**Antibodies**		

Alexa Fluor 647 anti-GFP Antibody	BioLegend	Cat#338006; RRID: AB_1279411

**Bacterial and Virus Strains**		

*Escherichia coli* strain DH5α	ThermoFisher	Taxonomy ID: 668369; Cat#18258012
*Escherichia coli* strain BL21	ThermoFisher	Taxonomy ID: 469008; Cat#C600003

**Chemicals, Peptides, and Recombinant Proteins**		

Super-folder green fluorescent protein	This study	N/A
Yellow fluorescent protein	This study	N/A
Monomeric enhanced green fluorescent protein	This study	N/A
mCherry fluorescent protein	This study	N/A
mVenus fluorescent protein	This study	N/A

**Critical Commercial Assays**		

HiScribe T7 High Yield RNA Synthesis Kit	New England Biolabs	NEB#E2040S
X-tremeGENE HP DNA Transfection Reagent	Merck	Cat#6366244001
Pierce BCA Protein Assay	ThermoFisher	Cat#23227

**Deposited Data**		

Subvolume averaging-derived signpost origami map	Electron Microscopy Data Bank	EMDB: EMD-12188
sfGFP-gB vesicle + SPOTs tomograms (Figure S4)	EMPIAR	EMPIAR: EMPIAR-10613
Atomic model of designed signpost origami	PDB	PDB: 7BHO

**Experimental Models: Cell Lines**		

HEK293T human embryonic kidney cells	ATCC	CRL-3216; RRID: CVCL_0063, female
DFJ8 chicken cells expressing mCAT-1	Gift from Walter Mothes (Yale University, USA)	N/A
BHK 21 baby hamster kidney cells	ATCC	ATCC® CCL-10; RRID: CVCL_1914, male

**Oligonucleotides**		

Staple oligonucleotides for signpost origami and SPOT construction: see supplementary Table S1	Integrated DNA Technologies	N/A
DNA templates for production of RNA aptamers for SPOTS: see supplementary Table S1	Integrated DNA Technologies	N/A
Oligonucleotides for adding fluorescence to SPOTs: see supplementary Table S1	Integrated DNA Technologies	N/A
Primers for insertion of fluorescent proteins in pEP98-gB: see supplementary Table S3	Integrated DNA Technologies	N/A
Primers for insertion of TEV restriction site as linker into mCherry-gB in pEP98-gB: see supplementary Table S3	Integrated DNA Technologies	N/A
Primers for ligation independent cloning of fluorescent proteins into pET-28, plasmid: see supplementary Table S3	Integrated DNA Technologies	N/A
Primers for ligation independent cloning of fluorescent proteins into pET-28, insert: see supplementary Table S3	Integrated DNA Technologies	N/A

**Recombinant DNA**		

Single-stranded scaffold DNA type p7249	tilibit nanosystems	M1-12
pET-28-sfGFP	This study	N/A
pET-28-mEGFP	This study	N/A
pET-28-YFP	This study	N/A
pET-28-mCherry	This study	N/A
pET-28-mVenus	This study	N/A
pcDNA3-MLV-YFP	Sherer et al., 2003	Gift from Walter Mothes (Yale University, USA)
pEP98-sfGFP-gB	This study	N/A
pEP98-mCherry-gB	This study	N/A

**Software and Algorithms**		

cadnano	Douglas et al., 2009b	https://cadnano.org/
CanDo	Castro et al., 2011	https://cando-dna-origami.org/
PEET (Particle Estimation for Electron Tomography)	Heumann et al., 2011	https://bio3d.colorado.edu/PEET/
IMOD	Kremer et al., 1996	https://bio3d.colorado.edu/imod/
UCSF Chimera	Pettersen et al., 2004	https://www.cgl.ucsf.edu/chimera/
FIJI	Schindelin et al., 2012	https://imagej.net/Fiji
SerialEM	Mastronarde, 2005	https://bio3d.colorado.edu/SerialEM/
MicroCal PEAQ-ITC Analysis Software	Malvern Pananalytica	https://www.malvernpanalytical.com/en/support/product-support/microcal-range/microcal-itc-range/microcal-peaq-itc/
Unblur	Grant and Grigorieff, 2015	https://grigoriefflab.umassmed.edu/unblur_summovie

### Resource Availability

#### Lead Contact

Further information and requests for resources and reagents should be directed to and will be fulfilled by the Lead Contact, Lindsay Baker (lindsay@strubi.ox.ac.uk).

#### Materials Availability

Reagents generated in this study are available on request from the Lead Contact with a completed Materials Transfer Agreement.

#### Data and Code Availability

The density map corresponding to the subvolume average of SPOTs has been deposited in the Electron Microscopy Data Bank (EMDB) as EMD: 12188 with the designed structure file PDB: 7BHO. The tomograms shown in Figure 4 have been deposited in EMPIAR as EMPIAR: 10613.

### Experimental model and subject details

#### Bacterial cells for cloning and recombinant fluorescent protein expression

*Escherichia coli* strains DH5α (Taxonomy ID: 668369) and BL21 (Taxonomy ID: 469008) were cultured in lysogeny broth (LB-Lennox) with 5 g/L NaCl at 37°C.

#### Cells for production of Murine Leukemia Virus

HEK293T cells (RRID:CVCL_0063, female) and DFJ8 (DF-1/J chicken cells, RRID:CVCL_XF11, sex unknown) expressing the gene for CAT-1 from *Mus musculus* (mCAT-1) [GenBank SLC7A1] were grown in Dulbeco’s Modified Eagle Medium (DMEM, GIBCO) with 1% GlutaMax (GIBCO) and 10% fetal bovine serum (FBS) (Sigma-Aldrich) at 37°C and 5% CO_2_. DFJ8 cells were a gift from Walter Mothes (Yale University, USA).

#### Murine Leukemia Virus source/genotype

The MLV genome was encoded on a pcDNA3 vector containing gagpol, gag, env-YFP sequences (Sherer et al., 2003). These plasmids were a gift from a gift from Walter Mothes (Yale University, USA).

#### Cells for expression of glycoprotein B

BHK 21 cells (RRID: CVCL_1914, male) were grown at 37°C with 5% CO_2_ in Glasgow Minimum Essential Medium (GIBCO) with 2%–5% FBS, 2% HEPES pH 7.2-7.5 (GIBCO), 2% tryptose phosphate broth (Sigma-Aldrich) and 1% GlutaMax.

HEK293T cells (RRID: CVCL_0063, female) were cultured at 37°C with 5% CO_2_ in DMEM supplemented with 1% non-essential amino acids (NEAA) (Sigma-Aldrich), 3% GlutaMax and 10% FBS.

All cell line details were accessed through Cellosaurus (Bairoch, 2018).

### Method Details

#### Aptamer production

##### Template Generation

DNA templates for aptamer transcription were purchased from Integrated DNA Technologies (4 nmole Ultramer DNA oligo, standard desalting). Sequences are provided in the Key Resources table below. Sense and anti-sense strands were mixed 1:1 in 100 mM Potassium Acetate, 30 mM HEPES, pH 7.5 at a final duplex concentration of 80 μM. Double-stranded template was prepared by heating the mixture to 94C° for 2 min, and cooling to 5°C at a rate of 1°C/minute.

##### Transcription

Transcription reactions were prepared using HiScribe T7 High Yield RNA Synthesis Kit (New England Biolabs) using the standard protocol for short transcripts. Duplex template was included at a final concentration of 2 μM, and the reaction mix was incubated for 16 h at 37°C. The transcription mix was diluted 1 in 5 in water, and template was removed by DNase I digestion. DNase I (New England Biolabs) was included at 0.2 units/μL in the supplied buffer and the reaction mix was incubated for 30 min at 37°C.

##### Purification

The aptamer was purified by ethanol precipitation. After DNase I digestion, 1 volume of unpurified aptamer was added to 3.5 volumes of ethanol and 1/8 volumes of 3 M sodium acetate. The ethanol precipitation mix was incubated at −80°C for 2 h and pelleted by centrifugation at 13,000 x g for 20 min at 4°C. The pellet was washed twice by addition of 70% ethanol and centrifugation at 13,000 x g for 10 min at 4°C. The pellet was resuspended in Tris-EDTA buffer, pH 8.0 (Applichem).

#### Origami construction with and without aptamer

##### Design and Materials

The signpost DNA origami structure was designed using CaDNAno (Douglas et al., 2009b). A model of the structure was generated using the CanDo web server (Castro et al., 2011). Single-stranded p7249 (M13mp18) scaffold was purchased from Tilibit Nanosystems. Staple strands were purchased from Integrated DNA Technologies (25 nmole DNA Plate Oligo, 96 well V-bottom plates, 200 μM in Tris-EDTA (TE) buffer pH 8.0). The strand Cy5_label was purchased from Integrated DNA Technologies (250 nmole Oligo, HPLC purification, dry) and resuspended at 200 μM in TE. The complete list of staple sequences required to fold the signpost origami structure is in Table S1. The mixes of staples required to fold different origami variants are given in Table S2.

##### Assembly

The one-pot assembly reaction mix was prepared as follows: scaffold p7249 (final concentration 40 nM) was mixed in Folding Buffer containing TE buffer and 12.5 mM MgCl_2_, with the appropriate staple strand mixtures (Tables S1 and S2) at final concentrations of 400 nM) and the aptamer (final concentration 4 μM). If used, the Cy5 or Texas Red fluorescent strand (5′-Cy5- or 5′-TexRd-XN/GGGAGTAGTAAGGAGGTGGT) is added at a final concentration of 9.6 μM with staple mix 5 (Table S2), which has extended sequences to hybridize with the Cy5 fluorescent strand in comparison to staple mix 4. The signpost DNA origami structures are folded by thermal annealing. The one-pot assembly mix is heated to 65°C for 15 min followed by cooling to 59°C at 1°C/5 min, cooling from 59°C to 35°C at 1°C/35 min, cooling from 35°C to 30°C at 1°C/10 min and cooling from 30°C to 15°C at 1°C/5 min. For checking folding by negative stain EM, ∼100 μL total reaction volume is used. To produce enough material for cryoET tagging, 1 mL total reaction volume is split into 10 × 100 μL to match wells in the thermocycler block.

##### Purification

Folded origami nanostructures were purified from excess staples by poly(ethylene) glycol (PEG) precipitation (Stahl et al., 2014). Unpurified origami structures were mixed 1:1 with PEG precipitation buffer (TE buffer, 12.5 mM MgCl_2_, 500 mM NaCl, 15% w/v PEG 8000) and pelleted by centrifugation at 20,000 x g for 30 min at 20°C. The origami pellet was then resuspended in 100 μL folding buffer (TE buffer with 12.5 mM MgCl_2_). The PEG precipitation step is repeated two more times, and the pellet is resuspended in Folding Buffer to the desired concentration.

Excess PEG may be removed by molecular weight cut-off (MWCO) filtration. For MWCO purification, the PEG-purified sample is supplemented with Folding Buffer to a final volume of 500 μL, applied to a 100 kDa Amicon Ultra-0.5 mL centrifugal filter device (Merck), and concentrated by centrifugation at 14,000 x g for 15 min at 20°C. 3 centrifugation steps are applied before recovering the sample, and the mixture is supplemented with 485 μL Folding buffer before each successive step. After centrifugal removal of PEG, initial volumes of 100 μL and 1 mL yield around 20 μL of origami at 100-200 nM and 1-2 μΜ respectively. For cryoET, concentrations of ∼1 μM are typically desired.

#### Fluorescent protein cloning

##### Primers, gene sequence, and plasmid for each construct

Synthetic DNA (GenScript) was purchased coding for the following fluorescent proteins: super-folder green fluorescent protein (sfGFP) (Pédelacq et al., 2006), monomeric enhanced GFP (mEGFP) (Zacharias et al., 2002), mVenus (Kremers et al., 2006), yellow fluorescent protein (YFP) (Ormö et al., 1996), and mCherry (Shaner et al., 2004). The genes were cloned into pet28a vector using Ligation Independent Cloning (LIC) (de Jong et al., 2006) and the primers in Table S3.

##### gB cloning

The original gB pEP98 Plasmid was a gift from G. Cohen (Penn State University). N-terminally labeled gB constructs were created by insertion of sfGFP or mCherry genes after the gB signal peptide (90 bp from start) using inserted EcoRI/XhoI restriction sites. The mCherry sequence was followed by a TEV cleavage site (ENLYFQS).

#### Biological sample preparation

##### Expression and purification of fluorescent proteins

The above plasmids carrying the genes for sfGFP, mEGFP, YFP, mVenus and mCherry fluorescent proteins with a N-terminal 6xHis tag were used to transform *E. coli* BL21 cells (New England Biolabs) by heat shock before plating on LB with ampicillin (AMP) at 50 μg/mL. Protein expression was performed by autoinduction, growing cultures at 37°C overnight using 5 mL overnight culture in 500 mL LB AMP supplemented with 1 mM MgSO_4_, 25 mM (NH_4_)_2_SO_4_, 50 mM KH_2_PO_4_, 50 mM Na_2_HPO_4_, 0.5% glycerol, 0.05% glucose, 0.2% alpha-lactose. Cells were harvested by centrifugation at 4,000 x g for 10 min at 4°C, and resuspended in 10 mL cold lysis buffer (20 mM Tris pH 7.4, 500 mM NaCl, 30 mM imidazole in H_2_O) with cOmplete EDTA protease inhibitor, following manufacturer’s instructions (Roche). Cells were lysed in a constant flow cell disruptor (Constant Systems) at 8,000 psi with lysozyme and DNase. Cell debris was removed by centrifugation at 21,000 x g for 30 min and the supernatant was bound to 5 mL Ni-NTA resin (QIAGEN) per liter culture for 60-90 min at 4°C. Resin was washed using 4 column volumes (CV) of wash buffer (20 mM Tris pH 7.4, 500 mM NaCl, 30 mM imidazole) and eluted with wash buffer containing 400 mM imidazole and 10% glycerol. The eluent was then run on a Superdex 75 300/10 GL size exclusion column (GE Lifesciences), and eluted in 0.25 mL fractions in PBS (10 mM phosphate buffer pH 7.4, 137 mM NaCl, 2.7 mM KCl). Six fractions were pooled for each protein and concentrated with centrifugal devices (Millipore). The protein concentration was estimated using the BCA assay (Pierce).

##### YFP-Murine Leukemia Virus production and purification

YFP-MLV was produced as described previously (Riedel et al., 2017). Briefly, live MLV virus stocks were prepared by transfecting HEK293T cells with a pcDNA3 vector containing gagpol, gag, env-YFP sequences (Sherer et al., 2003) (a gift from WM) using Fugene Xtreme Gene (Roche) as per manufacturer’s instructions. The cell supernatant was harvested after 5 days, cleared by centrifugation at 3000 x g for 5 min and stored at −80°C. The livevirus stock was used to infect DFJ8 in a T-25 flask (Cellstar) which was expanded to 6 T-175 flasks. Initial virus harvest was performed after 3 days, followed by 2 more harvests every 2 days. The virus-containing supernatant was precleared at 3000 x g for 30 min and centrifuged through a cushion of 15% sucrose in 20 mM HEPES pH 7.5, 150 mM NaCl and 3.6 mM CaCl_2_ (1/6 of total centrifuge tube volume) at 100 000 x g for 1 h at 4°C. The supernatant was aspirated, and the virus pellet was allowed to dissolve overnight at 4°C in a small volume of 20 mM HEPES pH 7.5, 150 mM NaCl and 3.6 mM CaCl_2_. The concentrated virus (0.5 mL) was then centrifuged at 15,000 x g for 1 min to remove large clumps and passed through Capto™ Core 700 1 mL pre-packed column (GE Healthcare) before snap-freezing in liquid N_2_ and storage at −80°C. YFP-Env for gel shift assays was purified using a method adapted from (Sjöberg et al., 2014). Briefly, virus particles were solubilized in Buffer A (20 mM HEPES,130mM NaCl, 3.6 mM CaCl_2_) containing 1M NDSB-201, 1% Triton X-100 for 1 h at 4°C followed by centrifugation at 25,000 x g for 20 min. The supernatant was concentrated using Vivaspin 1 kDa MWCO spin concentrators (ThermoFisher Scientific) and buffer exchanged using Zeba spin desalting column (ThermoFisher Scientific) into Buffer A.

##### SfGFP-gB and mCherry-gB expression for vesicle samples

Vesicle preparations were performed in a similar manner to Zeev-Ben-Mordehai et al., 2014a. In detail, HEK293T cells were grown to confluency in T175 flasks using supplemented DMEM (1% non-essential amino acids (NEAA) (Sigma), 3% GlutaMax (GIBCO)) and 10% FBS (Sigma). Transient transfection was performed using 30 μg DNA of respective gB constructs and 185 μL polyethylenimine (PEI), each diluted in 4.5 mL supplemented, serum free DMEM which were mixed and added to cells in addition to 9 mL supplemented DMEM with 4% FBS. After 24 h the medium was exchanged for 18 mL supplemented, serum-free DMEM and cells incubated for another 24 h. 48 h post transfection cell supernatant was collected and replaced with 18 mL supplemented, serum-free DMEM. Supernatants were harvested every 24 h up to 120 h post transfection.

Vesicles were purified from cell supernatant by centrifugation at 4,000 xg for 20 min at 4°C to remove cellular debris. The pellet was discarded and the supernatant loaded in SW32 ultracentrifugation tubes before being underlaid by 5 mL of 20% sucrose solution in HEPES buffer (130 mM NaCl, 20 mM HEPES pH 8). After a 2 h spin at 30 000 rpm (∼150 000 xg) in a SW32 rotor (Beckman Coulter) at 4°C the supernatant was completely removed and discarded. Pelleted vesicles were rehydrated overnight in a small volume of HEPES buffer (100 μL / T175 flask).

##### BHK 21 growth on grids and sfGFP-gB expression

Quantifoil 2/1 carbon film gold-mesh grids were plasma cleaned (Harrick Plasma) for 2 min and incubated in decanoic acid under a short-wave UV light in a class 2 biosafety cabinet overnight. Grids were then sequentially washed in 12 volumes methanol, ethanol, and sterile phosphate buffered saline (PBS) before storing each grid in 50 μL PBS in one well of 2x9-well microslides (Ibidi) at 37°C until use.

BHK-21 cells were grown to confluency in a T25 flask, before removal with trypsin and seeding onto the treated Quantifoil gold-mesh grids in fresh 2x9-well microslides (Ibidi). Grids were seeded at 2x the desired density to allow for cells adhering to the well surface. Cells were left to adhere overnight, and transfected the next day with the sfGFP-gB construct using the Xtreme-Gene HP transfection kit (Roche) with a DNA:reagent ratio of 1:3. Cells were incubated at 37°C for 8-14 h post-transfection before plunge freezing.

#### Binding and biophysical assays

##### Gel shift assays

Agarose gels: 100 nM YFP or Env-YFP were mixed 1:1 with Folding Buffer or 440 nM SPOTs. Samples were incubated for 15 min on ice and analyzed by agarose gel electrophoresis (2% agarose gel, 1x TAE buffer (Sigma-Aldrich), 11 mM MgCl_2_, run at 60V at 4°C for 2 h).

Polyacrylamide gels: 1 μM YFP, mCherry, mEGFP, sfGFP or mVenus were mixed 1:1 with Tris-EDTA buffer or 10 μM aptamer. Samples were incubated for 15 min on ice and analyzed by polyacrylamide gel electrophoresis (6% Acrylamide/Bis 29:1, 0.5 x TBE buffer (Sigma-Aldrich) at 180 V for 40 min.

Scanning conditions: Both agarose and polyacrylamide gels were scanned with an Amersham Typhoon Gel Imaging System (GE Healthcare). GFP fluorescence was imaged using a 488 nm laser and 525 nm bandpass filter combination. mCherry fluorescence was imaged using a 532 nm laser and 670 nm bandpass filter combination.

##### Isothermal titration calorimetry (ITC)

Fluorescent proteins and aptamer (variant without the 3′ extension for origami conjugation) were prepared in ITC buffer (PBS (ThermoFisher Scientific) + 5 mM MgCl_2_) by dialysis using 10K Slide-A-Lyzer MINI devices (ThermoFisher Scientific) at 4°C overnight. Protein concentrations were measured prior to dialysis by Pierce BCA assay.

ITC experiments were carried out at 25°C on a MicroCal PEQ-ITC microcalorimeter (Malvern Panalytical). 1-3 μM aptamer was placed in the sample cell with a volume of 250 μL and binding isotherms were recorded following injections of ∼30 μM fluorescent protein (4 μL each).

##### Bio-layer interferometry (BLI)

BLI measurements were performed on the Octet system (ForteBio). Briefly, amine reactive group 2 (ARG2) tips (ForteBio) were pre-soaked in buffer FPO (PBS + 12.5 mM MgCl_2_) before activation with equal parts 20 mM N-hydroxysuccinimide (NHS) and 200 mM 1-ethyl-3-(3-dimethylaminopropyl)-carbodiimide (EDC) for 5 min, followed by a 5 min incubation with buffer FPO, purified fluorescent proteins or vesicles, quenching in 1 M Tris pH 7.4 for 5 min, and washing with buffer FPO. Example tip loading curves are shown in Figure S2. After loading, tips were stored at 4°C in buffer FPO until use later the same day. SPOT binding was assayed by equilibrating the tips in buffer OS (TE + 12.5 mM MgCl_2_ +100 mM NaCl) for 3 min, then measuring association of the appropriate SPOT or origami without aptamer (both at ∼100 nM) for 8-10 min, followed by a 8-10 min dissociation measurement in buffer OS.

##### Fluorescence microscopy

HEK293T cells were grown to confluency in a T75 flask, before removal with trypsin and seeding into 18-well chambered coverslips coated with ibidiTreat (Ibidi) at a density of 3x10^5^ cells per well. Cells were left to adhere overnight, and transfected with the sfGFP-gB construct (100 ng) using the Xtreme-Gene HP transfection kit (Roche) with a DNA:reagent ratio of 1:3 (μg:μl) at 37°C for 20 h. Post-transfection the cells were washed once and incubated in Fluorobrite (GIBCO) with 1% GlutaMax (GIBCO) and 10% FBS (100 μL per well) for 1 h. Culture media (100 μl) was supplemented with a final concentration of 5 mM MgCl_2_ before 1 μL of 100 nM aptamer origami tagged with cy5 was added directly to each culture well and incubated for ≳15 min before imaging. Live cell fluorescence microscopy data were recorded on a SP8 X-SMD FLIM confocal microscope (Leica). Images were recorded at 63x magnification and processed in FIJI (Schindelin et al., 2012).

##### Dynamic light scattering (DLS)

DLS measurements were acquired on an Uncle System instrument (Unchained Labs). SPOTs were prepared in folding buffer (TE buffer with 12.5 mM MgCl_2_) at 2x the required concentration and diluted to 200, 100, 50 and 10 nM by the addition of equal volumes of buffer (PBS with 5 mM MgCl_2_), or culture medium (DMEM with 10% FBS and 5 mM MgCl_2_). The positive control aggregated SPOTs were produced by skipping the final temperature decrease (30°C – 15°C) in the folding protocol and selecting for large aggregates (following one round of PEG precipitation to remove excess staples) by spinning the sample without PEG, removing the supernatant and resuspending the pelleted material. 9 μl of these samples including buffer and culture medium baseline controls (50:50 mixture of folding buffer and either PBS with 5 mM MgCl_2_ or DMEM supplemented with 10% FBS and 5 mM MgCl_2_) were loaded into a UNi and 10 DLS acquisitions of 10 s each were measured at 25°C.

#### Sample preparation and electron microscopy

##### Negative stain EM of signpost origami

For negative stain TEM imaging, 10 μL of a 5 nM origami sample solution was applied to glow-discharged Carbon type-B-Formvar supported copper grids (Agar scientific) for 2 min. The sample was subsequently stained using a solution of 2% uranyl acetate for 10 s. Negative stain EM data were recorded on a T12 microscope (FEI, Thermofisher) operating at 120 kV, equipped with a OneView camera (Gatan). Images were recorded at 30,000x nominal magnification and processed in FIJI (Schindelin et al., 2012).

##### Incubation of SPOTs with biological samples

sfGFP-gB vesicles or MLV preparations were mixed 1:1 with aptamer-origami (final concentration ∼200 nM) and incubated on ice for 30 min. For cellular samples, the 50 μL of culture medium surrounding each grid was supplemented to a final concentration of 5 mM MgCl_2_. 1 μL of 200 nM aptamer origami was added directly to each culture well and incubated for ∼15 min before vitrification. Longer incubations could lead to issues with endocytic recycling of membrane proteins with bound SPOTs or degradation of the RNA aptamer by RNases in FBS that has not been heat inactivated.

##### Plunge freezing

4 μL of vesicles or virus were applied to a freshly plasma-cleaned (Harrick Plasma) Quantifoil 2/1 holey carbon copper-mesh grids (Agar Scientific). Grids with adherent cells were removed from the culture medium immediately prior to plunging. 1 μL of 10 nm fiducial gold markers (Aurion) were added to the drop on the grid for each sample before blotting away excess by hand and plunging into a reservoir of propane/ethane with a manual plunger. Grids were stored under liquid nitrogen until imaging.

##### CryoEM

CryoEM data were recorded on either a TF30, TF30 Polara or Titan Krios microscope (FEI, Thermofisher), equipped with K2 or K3 direct electron detectors and (Bio-) Quantum energy filters (Gatan). Tomographic data was collected with SerialEM (Mastronarde, 2005) with pixel sizes between 1 and 3 Å/pixel at the specimen level and the energy-selecting slit set to 20 eV. The sfGFP-gB vesicles (±60°) and MLV (±66°) datasets were collected using dose-symmetric acquisition and the cell data (±60°) was collected using bi-directional acquisition schemes (starting from −21° up, and returning to −24° to collect the negative tilts) with 3° tilt increments. 7-15 image frames (0.2 s exposure/frame) in counting mode were collected per tilt at a dose rate of ∼5 e^−^/unbinned pixel/second, giving an overall dose of between 100 and 225 e^−^/Å^2^. Defoci between 2 and 5 μm underfocus were used to record tilt series and projection images.

##### Tomogram reconstruction and data processing

Frames were aligned and filtered for radiation damage using Unblur (Grant and Grigorieff, 2015) for the sfGFP-gB vesicles, and on the fly in SerialEM for the other datasets. Tomograms were reconstructed using IMOD (Kremer et al., 1996). Contrast transfer functions were measured and data phase-flipped in IMOD. Tomograms were further processed for viewing in FIJI (Schindelin et al., 2012). Segmenting was done manually in FIJI. Sub-volumes were picked manually in IMOD and sub-volume averaging and classification was done with PEET (Heumann et al., 2011). Segments, sub-volume averages and atomic models were visualized with UCSF (University of California San Francisco) Chimera (Pettersen et al., 2004). Template matching was done with PEET, using the subvolume average of the DNA origami template derived from Figure 2D as the template, and with the ‘no reference refinement’ option switched on. The initial particle model was a grid of points with 15 Å spacing.

### Quantification and Statistical Analysis

#### Isothermal titration calorimetry

Data were processed and analyzed using a non-linear least-squares fit to a one-site binding model using MicroCal PEAQ-ITC Analysis Software (Malvern Panalytical). The binding stoichiometry and protein concentrations were specified in the fit while the thermodynamic parameters and aptamer concentrations were varied, as aptamer concentrations measured from absorbance were inaccurate due to residual rNTPs. The fitted aptamer concentrations were consistent across all ITC experiments, allowing for dilution effects.

#### Bio-layer interferometry

The raw data were exported from the interferometer software (ForteBio) and processed in Excel (Microsoft). The unloaded buffer controls were subtracted from each experiment, and the magnitude of the binding scaled by the amount bound to the tips, as measured by the final wash value during tip loading. With the exception of the titration curve (Figures S6A), each measurement was made in triplicate and the results averaged to ensure any variation due to the heterogeneous sample nature was minimized. For the titration curve and rigid SPOT binding assays, the curve for 100% mCherry vesicles was used as a further baseline after buffer subtraction. For rigid SPOT binding assays, the maximum amplitude of each ‘normal’ SPOT binding was set to 1 to aid in comparison.

#### Dynamic light scattering

The raw data were exported from the instrument software (UnChained Labs) and processed in Excel (Microsoft). The instrument software applies a scaling regime which means raw intensities are unavailable for comparison across samples, so the amplitude of the signal from samples with concentrations lower than 200 nM were scaled by the ratio of the samples derived intensity (in counts per second) to the 200 nM sample’s derived intensity.

#### Sub-volume averaging and template matching

Sub-volume averaging was done using PEET (Heumann et al., 2011). For the origami-only averaging, sub-tomograms of 694 origami particles from 54 tomograms were selected manually. An initial orientation was assigned to each sub-tomogram, with the y axis directed along the nanostructure ‘post’. An initial average was created using these rough orientations and used as the first reference in the alignment. The whole dataset was used for iterative sub-volume refinement. For the averaging of the origami together with membrane-bound gB, 661 particles were selected from 54 tomograms, with each particle’s y axis pointing to the center of the vesicle to which it is bound. We attempted to use template matching to automatically identify SPOTs in these tomograms, using the subvolume average from Figure 2D as a reference. Unfortunately, of all the conditions tested, the best identified only 60% of the manually picked SPOTs, with a false positive rate > 65% for all particles. To identify 90% of the manually picked SPOTs (the maximum number identified), the required conditions produced a false positive rate of nearly 80%. As excluding the false positives required more time than manual picking, we concluded that template matching requires further development for use with SPOTs.

As above, an initial average was created and used for subsequent refinement. No mask was applied during this process. PCA-based classification was then applied to the resultant particles to create 4 classes, with the goal of mitigating the effects of flexible linkage between gB and the origami structure while maintaining enough particles per class to produce viable averages. No mask was applied during this process. Visualization of the reconstructed volumes was performed in USCF Chimera (Pettersen et al., 2004).

## Supplemental information

Document S1. Tables S1–S3

Table S4. Microsoft Excel file for purchasing required staple strands from commercial oligonucleotide suppliers, related to Table S1 and STAR methods

Video S1. Slicing in z through a tomogram of SPOTs bound to YFP-tagged murine leukemia virus (related to Figure 5) Slice thickness 0.5 nm.

Video S2. Slicing in z through a tomogram of SPOTs bound to BHK-21 cells expressing sfGFP-gB (related to Figure 6Ci) Slice thickness 2.8 nm.

Video S3. Slicing in z through a tomogram of SPOTs bound to BHK-21 cells expressing sfGFP-gB (related to Figure 6Cii) Slice thickness 2.8 nm.
