# Supplementary material for: DNA origami signposts for identifying proteins on cell membranes by electron cryotomography
Source: Cell. 2021 Feb 18;184(4):1110–1121.e16. doi: 10.1016/j.cell.2021.01.033 (PMC7895908; doi:10.1016/j.cell.2021.01.033)
Supplement: Document S1. Tables S1–S3 [file mmc1.pdf]

Cell, Volume *184*

Supplemental Information

DNA origami signposts for identifying proteins

on cell membranes by electron cryotomography

Emma Silvester, Benjamin Vollmer, Vojtěch Pražák, Daven Vasishtan, Emily A. Machala, Catheryne Whittle, Susan Black, Jonathan Bath, Andrew J. Turberfield, Kay Grünewald, and Lindsay A. Baker

Table S1 –List of oligonucleotide staples required to construct SPOTs and their precursors or derivatives, related to STAR Methods. This table is also available as a CSV file for ordering from commercial oligonucleotide suppliers (Table S4). All oligonucleotides require a standard desalting purification unless indicated.

Name	Sequence	Mix	Notes
**Staple Oligonucleotides**			
A-0[111]16[102]	TCGCCCAGCAGGGAGACTTTTCAACATTGAAAGATTCA	1	25 nmol
A-0[195]4[182]	TTGTATCGGATTTTCATAGTTAGCGTAAACCCTCATAGGTGT	1	25 nmol
A-1[116]67[121]	AAGCATCTTTAATCACCGCCTTAACAATTTTTCAATATATATCTTTAG	1	25 nmol
A-1[140]5[149]	CCCTCAGGATACCGAGTAACAGTG	1	25 nmol
A-1[163]3[174]	ACGTTGGTGAATAGTTAATGCCCCCTTACTCAGCCCTCAG	1	25 nmol
A-1[203]3[216]	AACTTTCAAAGGAGCATGAAAGTATTAAAGAGGGTGGGATAG	1	25 nmol
A-1[85]13[90]	TTTTTCGGTCGCTTAGTAAATTGGGCTGAATTAC	1	25 nmol
A-10[106]66[92]	CCAGCGGATATTCATTGTCATTTCTTAGAGTGAGTAACCGAAC	1	25 nmol
A-10[136]66[130]	CCACGCCTTTAGCCATATGGAGCGGAGACTTTACA	1	25 nmol
A-10[166]31[166]	TTTTCCTCAGAACTAAAAACAGTTTT	1	25 nmol
A-11[105]6[98]	ACATCAATCATACAGAACGAGGAGGCTTCGCATAAGTGTCGACTGCTCC	1	25 nmol
A-11[126]67[139]	GAGCCACCACCGGACGACTTGCAACGCTCCTCAAAACTAATA	1	25 nmol
A-11[64]67[90]	TTTTGCTCATTCAGATTCCCGCAACTATCGACTCAGTGCCAAGCTTGCATAAAGG	1	25 nmol
A-12[134]5[125]	TTTTATTAGCGTTTGCGCCGCTTAATGACATTTAACG	1	25 nmol
A-13[64]69[95]	TTTTCAACTTTAAATGGTCATTTGACCCCATTAACGAACCACCAGCAGAAGATAAAACAG	1	25 nmol
A-13[91]13[111]	CTTATGCGATTTTAAGGGCAT	1	25 nmol
A-14[111]51[101]	TCATCAACTGGCTCAACGGAATCATGAGGCCACTAAACGGAGTAGCACCCACCGACCAGT	1	25 nmol
A-14[127]13[134]	TTTTTGTAGCGCGTTTTTTCGGTCATAGCCCCCTTTTTT	1	25 nmol
A-14[90]40[81]	CAGTCAGGAATTAGCAATAAAGCC	1	25 nmol
A-15[68]15[90]	TTTTAAAAATCTACGTTAATAAA	1	25 nmol
A-16[101]17[127]	TCAGGAATTACGAGGCAGAATCAAGTTTGCTTTT	1	25 nmol
A-16[127]15[127]	TTTTCTTTAGCAGGTAATTACGTCAGACTTTT	1	25 nmol
A-16[90]42[81]	TTAGGAAGAAGCCTGCAAGGATAA	1	25 nmol
A-18[127]20[98]	TTTTTAATCAGTAGCGACATAGTAAGAGCAAGGCTTTGTTTTGC	1	25 nmol
A-19[68]20[77]	TTTTACGACGATAAAAGTAAAAT	1	25 nmol
A-2[132]21[131]	ACAGAGGCTTTGAGTAAAACGACCAAGCGGCCGGAAATTTT	1	25 nmol
A-2[143]10[137]	TAGCATACACTTCTTTGACCCCCAGTGCCTTGATAGTTGGCAGGTCCCA	1	25 nmol
A-2[167]1[162]	GTCGTCTTTGCATCAGACACCAG	1	25 nmol
A-2[209]0[196]	CCACAGAATTTTCATGATATAAGTATAGTCTGAAACCTTTAA	1	25 nmol
A-20[131]19[127]	TTTTCGTCACCAATGAAACCATAAGAATGCAACGATAGCAGCACCGTTTT	1	25 nmol
A-21[84]45[107]	AGCAAAAGGTAATAACCAAAATAAAGTACAGCTAATGCAGAACGCGCTTTTT	1	25 nmol
A-22[131]51[131]	TTTTCATTAAAGGCAGTAGGGCTTTT	1	25 nmol
A-23[93]7[111]	TGAGGATGTTACCTTTGAAAGAGGAGTAAGCGT	1	25 nmol
A-23[98]95[107]	GAGGGAAAAGGGCGTTAAATATACCGACTGCCGTAAAG	1	25 nmol
A-24[131]53[131]	TTTTTGGTTTACCAATCATAATTTTT	1	25 nmol
A-24[92]53[83]	CAACTGCTTTAAACAGAAGATTG	1	25 nmol
A-25[112]59[127]	CAATAGAAAAGAAAACATACAGAATACCGATGATGAAACAAATCAATATCGATAGCTT	1	25 nmol
A-25[54]51[69]	AATCAAAAATTCGCGTTAAATCAGCTCATTACGTTAAGCCCCAATGTACCCAGAGAAT	1	25 nmol
A-25[86]26[90]	TAGTCAGAAGTTGGAATAAG	1	25 nmol
A-26[104]55[107]	ACACCACTATTTTGCAAATCCTCAAATATAT	1	25 nmol
A-26[131]25[131]	TTTTCAACATATAAAATTCATATTTT	1	25 nmol
A-26[89]6[81]	CAAAGCTGACCAATTAGCCGGAA	1	25 nmol
A-27[50]57[65]	TTTTCGAAAGACTGGGAACAATCAACATTA	1	25 nmol
A-27[77]62[91]	TTAATTTACGCAGTATGTTAGTGATTAATCAATTAATCGCGC	1	25 nmol
A-28[142]62[136]	AACCGAGGAAAAAACAAAATA	1	25 nmol
A-28[166]61[166]	TTTTGAACAAAGTAACAATTTCTTTT	1	25 nmol
A-28[85]61[76]	CTTATCGAGCTTCAAATCGCACT	1	25 nmol
A-29[147]2[144]	AAGTAAGAATCCTCTGGCCTTACAGCTTCAGCGAAGGAACGAGGG	1	25 nmol
A-29[54]29[83]	TTTTGGAAGCAAACTCCATTGAGTTAAGCC	1	25 nmol
A-29[84]9[93]	CAATAATTGTACAGGAGTAATCTT	1	25 nmol
A-3[162]4[156]	CCGCCAGAGGTTTAGTAC	1	25 nmol
A-3[175]5[188]	AACCGCCCGATCTAGAATTTTCTGTATGGGTTTATTCGGAAC	1	25 nmol
A-3[78]43[107]	TTTTGGGTAAAATCATAACCCATAACGAATAGATACCAATCAATAATCGGCTGTTTTT	1	25 nmol
A-30[104]57[108]	GAGCGCTAGAAACAACTGGCACAAACGTAATAGTGCTTTTTAACCT	1	25 nmol
A-30[153]11[166]	TTAGACGAATAACACGCCACCCCCTCAGAGCCGCCACTTTT	1	25 nmol
A-30[166]63[166]	TTTTGGAAGCGCAACAGTAACATTTT	1	25 nmol
A-31[54]65[76]	TTTTGAGAGTACCTTTAATTGCTATAATGCATGTGCT	1	25 nmol
A-31[84]11[104]	ATAAGAGACCCAAAAGGCTTGCCCTGACGAGAAAC	1	25 nmol
A-31[98]10[107]	TTGCGTTTTGTTTACACCA	1	25 nmol
A-32[125]27[131]	CCCAATCAAATGAAGAACAAAGCTATCTAATAACGTAAAGGTGGTTTT	1	25 nmol
A-32[166]65[166]	TTTTATTTGCCAGTCCTGATTATTTT	1	25 nmol
A-33[105]35[115]	CCAGCTAATCAAGATACCGCGCCCA	1	25 nmol
A-33[54]34[54]	TTTTTGTTTTAAATATAATTCTGCGAACTTTT	1	25 nmol
A-34[153]35[166]	TTTAGCGCCGGTATTCTAAGAACTTTT	1	25 nmol
A-34[166]33[166]	TTTTGCGAGGCGTCAGAGCCTATTTT	1	25 nmol
A-34[99]8[77]	TGCTTTCATTCCATATAACAGTTGTGAATATCAACGTTCATCAAACCAGGC	1	25 nmol
A-35[116]7[131]	ATAGCAAGCATTTTGAAGGAACCAGCCGCCAGACAGGAGCAGTCTGGCTTTTGATTTT	1	25 nmol
A-35[133]8[143]	TATAGAAGGCTTATAACCTCCACCGCCTCTCAGAGAGACGATATTA	1	25 nmol
A-35[54]36[54]	TTTTGAGTAGATTTAGATAACCTGTTTATTTT	1	25 nmol
A-35[83]34[100]	TACTTTTCATCGTAGGAATCATTTAGT	1	25 nmol
A-36[104]36[81]	TTTTTGCAAGCCGTTTTTAATTTC	1	25 nmol
A-37[54]76[63]	TTTTGCTATATTTTCATCTACTATTGCGCTTTTCCAG	1	25 nmol
A-37[83]38[81]	CTGCTCATCCGCAAAAAG	1	25 nmol
A-38[107]37[104]	TTTTTTTAAACCAAGTACGAGAACAATTTTT	1	25 nmol
A-39[47]78[63]	TTTTTTAACATCCAATAAATCATAGCTAAACGGGGAGCGTATTG	1	25 nmol
A-4[111]1[115]	AAAGTACCGAAGGCACCAACCGACTAAAGTTA	1	25 nmol
A-4[155]3[161]	CGCCACTCAAAAACACCCTCAGAA	1	25 nmol
A-4[181]2[168]	ATCACCGGCCTATTCAGCTTGCTTTCGAAGTAAATAAGTTTT	1	25 nmol
A-4[222]2[210]	CCGTCGGAGGCTGAAAAAAAGGCTCCAAACAGTTTAGCATT	1	25 nmol
A-40[107]39[107]	TTTTTTCCAAGATTAAGCAAAAACGGGTATTTTT	1	25 nmol
A-40[76]71[92]	AGCATAAACAGGCACATCAATTTTGGGGGAATGGCTAATGCGCGAACTGATAGCTTTT	1	25 nmol
A-41[47]41[76]	TTTTAAAACATTATGACCCTGTAATACTTT	1	25 nmol
A-42[107]41[107]	TTTTTTCTTTCCTCAACTTATTTTATCATTTTTT	1	25 nmol

A-42[72]80[66]	AGAACCCTCAGATTGCCTGGC	1	25 nmol
A-42[80]47[102]	AAATTGTAGAAAAGTCCTACATGTTCCGACAAACAGTCAAATCAATTTTCGAGCCAGT	1	25 nmol
A-43[47]44[47]	TTTTGCAATGCCCATCATATCCTGAGTATTTT	1	25 nmol
A-44[107]14[91]	TTTTTCTGTTTATCAACCCAAAAGTTGAGATACGAACTATTATAC	1	25 nmol
A-44[69]81[85]	AAAAATACTAATTTCCTACATACATTGGCAGATTCACCAGTTTT	1	25 nmol
A-45[47]46[47]	TTTTATGTGTACAGACCTGTCGGTAAAGTTTT	1	25 nmol
A-46[107]18[95]	TTTTTAATAAGAGAATATAGCGAGACA	1	25 nmol
A-46[72]84[63]	TAAAGTAATTGACGACAGGCCTTG	1	25 nmol
A-47[47]48[50]	TTTTATTCAAAAGGGTAGCTGATAATTTT	1	25 nmol
A-48[100]89[89]	TTTTTCAGAGGCCCATCAATATGAGTTTTTATATTTT	1	25 nmol
A-49[50]50[50]	TTTTATTAATGCCGAGAGTCTGTTTT	1	25 nmol
A-49[77]49[100]	TTTTTGAGAGATATTTAGGTTTTT	1	25 nmol
A-5[114]66[109]	TAAGTACAACCACAGCATTCCAGAACACGTCAACAAATAAGGAACAAAACTCGTATT	1	25 nmol
A-5[126]1[139]	GGGTCAGCGATTATAAAGAGGCAAAAGAAACGGCTATCGTCA	1	25 nmol
A-5[150]9[166]	CCCGTATAAACTTCTTAAGATATTCACTTTT	1	25 nmol
A-5[189]1[202]	CTATTATCCCGGAAGAGCCACCACCCTCCAGCCCTGCTAAAC	1	25 nmol
A-50[131]6[115]	TTTTTTAATTGAGAATCGCCCGCTCAATGAATTAATTGACGAGTG	1	25 nmol
A-50[69]85[85]	ATTGCCTGGAGAGGCCGTTCTGAGAAAGTAACCGTCACTTGCCTGAGTAGAAGATTTT	1	25 nmol
A-50[73]51[92]	GGTCCGATGAACGGTAATCGTAAAATT	1	25 nmol
A-50[97]91[89]	CAACATGTACTACAAAGGCTAAGGAGGCCGTTTT	1	25 nmol
A-51[102]94[91]	ATATCATATGGCTGCGCACGGGGAAAGCCGG	1	25 nmol
A-51[50]52[50]	TTTTGAGCAAACACGGTTGATATTTT	1	25 nmol
A-51[93]22[84]	CTTACTTGAGCCATTTGGG	1	25 nmol
A-52[131]93[131]	TTTTTACTAGAAACGCCGCGCTTTTT	1	25 nmol
A-52[92]94[77]	TACAAACTAGCATGTCGCTGGCAGGCGAGA	1	25 nmol
A-53[50]95[65]	TTTTATCAGAAAATATTTTGCTACGTGAAC	1	25 nmol
A-53[84]24[93]	TATAAAATAAGGCGACATT	1	25 nmol
A-54[114]60[105]	TGGGTTAATTTGTAAATGCTTAGGCGCTGAGAACATAGATGTGAGTGAATAA	1	25 nmol
A-54[131]95[131]	TTTTGACCTAAATAACCCTAAATTTT	1	25 nmol
A-54[92]95[83]	GATAGCAAATATTTAATCAAGTT	1	25 nmol
A-55[108]50[98]	TTTATTTGAAAAGAATAATTTAGTAAAGCCAAATATTTAACAACGC	1	25 nmol
A-55[40]56[40]	TTTTTTAAATTTTTGTCTGGCCTTCCTGTTTT	1	25 nmol
A-55[87]59[97]	AGAAAACTTTTAATCGCAAGACTACAATTTATTTTTCCC	1	25 nmol
A-56[131]55[131]	TTTTATAACTATATCATCTTCTTTTT	1	25 nmol
A-56[85]57[76]	AAGAGGAACGCCATCATGAGCGA	1	25 nmol
A-57[109]4[112]	CCGGCTGATGTCACAATAAGACAAGGTAAATTCACCGTATTACCATTAGCAAGCGAAAC	1	25 nmol
A-57[40]58[40]	TTTTTAGCCAGCTTTCAACGGCGGATTGTTTT	1	25 nmol
A-57[66]24[50]	AATGAAAATAATCAGGTCAACGAGAATGACCATATTTT	1	25 nmol
A-57[77]56[86]	GTAACAGGTCTGAGAGACA	1	25 nmol
A-58[131]57[131]	TTTTAGATTAAGATTGGGTTATTTTT	1	25 nmol
A-58[85]59[76]	TCATAACCCGTCGGATGTAGATG	1	25 nmol
A-59[40]60[43]	TTTTACCGTAATGGGAAGGGGACGATTTT	1	25 nmol
A-59[66]26[50]	TGGTTCTCCGTTCAAATAAGATTAAGAGGAAGCCTTTT	1	25 nmol
A-59[77]58[86]	GGCGCATTAATTAACAAAA	1	25 nmol
A-6[114]5[113]	TACTGGCGACAATCCGTAA	1	25 nmol
A-6[131]23[131]	TTTTTGATACAGGGAAATTATTTTTT	1	25 nmol
A-6[80]23[92]	CGAGATCCCCCTCAAACGAT	1	25 nmol
A-60[104]26[105]	CCTTGCTTTAGAATCCTTGAAAAGAGTCAGAAAATCGCAAAG	1	25 nmol
A-60[135]1[132]	AGTACATAAACATCAAGACGCAATTACCGAAGGAAAGCGGTTGAGCGCCGACTTGCGGG	1	25 nmol
A-60[166]28[147]	TTTTATTTGAATTACCTTTTTACATTTTACCAGA	1	25 nmol
A-60[69]28[54]	CATCTGCAGGAAGAGCGAACCAGACCTTTT	1	25 nmol
A-60[97]60[70]	TCTGTAAATCGTCGCTATCGTAACCGTG	1	25 nmol
A-61[105]8[98]	AAAAGAACAAAAGAATGAAATTACCGTTCCACAGA	1	25 nmol
A-61[43]62[43]	TTTTCGACAGTATCGGAAACCATTTT	1	25 nmol
A-61[77]28[86]	CCAGCTTATTCATTGACTC	1	25 nmol
A-62[114]30[105]	GAATACCAAGAGAAATAGGTAATT	1	25 nmol
A-62[135]10[126]	ACGGATTCGCATTTTCAACACCCTAATAGCACCTCAGA	1	25 nmol
A-62[166]63[153]	TTTTGTACCTTTTACATCGGTGAATAT	1	25 nmol
A-62[90]63[69]	AGAGGCGAACAGCTTTCCGGCACCGCTTCCATTCA	1	25 nmol
A-63[43]64[43]	TTTTGGCAAAGCGTCTTCGCTATTTT	1	25 nmol
A-63[70]30[84]	GGCTGCGCAACTTCAAAATTAGAGAGAT	1	25 nmol
A-64[108]32[98]	ATAAATTTTGCGAAACGATTGATGG	1	25 nmol
A-64[139]35[132]	ATAATCCTGATTGTACCAGAATATTTATATCTTACCGGGAGGAATCAGA	1	25 nmol
A-64[166]33[153]	TTTTTCAGATGATGGCAATTATCATATTTACAAAGTCTTTC	1	25 nmol
A-64[62]59[65]	GCGGGCCCCATTCGCTGGTGCCGGCCTCCAGTTTGTAGGTCACGT	1	25 nmol
A-64[69]32[54]	GATCGGTAAGGGGGTGTAGCTCAACATTTT	1	25 nmol
A-65[43]66[43]	TTTTTTACGCCAGTTTCCCAGTTTTT	1	25 nmol
A-65[86]33[104]	AAGTTCTTAATTGTGTCTGGAAGTATTTTGCAC	1	25 nmol
A-66[108]69[115]	AAATATCTAAACTGGTCAGGTCAGTATTA	1	25 nmol
A-66[129]69[136]	AACAACTAACATATCAAACAACAGTGCCA	1	25 nmol
A-66[166]67[150]	TTTTACATTTGAGGATTTAGGCCG	1	25 nmol
A-66[76]67[69]	TAAGTTGGGTAACGGACGGCC	1	25 nmol
A-66[91]65[85]	GTTATTAATTGCGATGCAAGTTAA	1	25 nmol
A-67[122]62[115]	GAGCATTCGACAGAAACCTTGGATTTGCGTAGCTGATTGCTTT	1	25 nmol
A-67[140]60[136]	GATTAGAAAGTATTAATTATCCATCAATCGTCAGAGAGAAACAATTAATTTAATGGAAAC	1	25 nmol
A-67[151]69[166]	TCAATCACCTCAGCAGCAAATGAAAATTTT	1	25 nmol
A-67[43]65[62]	TTTTCACGACGTTGTAAAACCCAGGGTCTGGCGA	1	25 nmol
A-67[91]61[104]	AATTGAGGAAGGTTCCTTTGCCATTATCTGGAAGGGTAAAACTTACAAACCTGAGC	1	25 nmol
A-68[166]67[166]	TTTTATCTAAAGCATAGATAATTTTT	1	25 nmol
A-68[69]68[43]	TAGAGGATCCCCGGGTACCGAGCTTTT	1	25 nmol
A-68[81]30[54]	GCAGGAAGTACGGCTGAATCCTTTTGAACCCACAAGAAACAGGTCAGGATTATTTT	1	25 nmol
A-69[116]33[125]	ACACCGCCTGCCCTCAAATCCTGA	1	25 nmol
A-69[137]28[143]	CGCTGAGAGCTGCTGAAAACGAGCATAAACACAGAGAGGGAGAATTTAAGAAAGGA	1	25 nmol
A-69[43]69[69]	TTTTTCGAATTCGTAATCATGGCCGAA	1	25 nmol
A-69[96]68[82]	AGGTGAGGCGTTGGCAAATCAACAGTTGGCCT	1	25 nmol
A-7[112]64[109]	CATACATCTGAATTAGCAATAGTCAGAGAAGAAATATACTTCTGA	1	25 nmol
A-7[77]27[76]	ACCGAACGGATTGCATCAAAATCGCGTT	1	25 nmol
A-70[69]70[43]	AAATATCATAGCTGTTTCCTGTGTTTT	1	25 nmol
A-70[92]35[82]	TTTTCCTAAAACATCGATTAGA	1	25 nmol
A-71[43]71[69]	TTTTTGAAATTGTTATCCGCTCGTCTT	1	25 nmol
A-72[69]72[43]	TATTAACAATTCCACACAACATATTTT	1	25 nmol
A-72[92]37[82]	TTTTGACAATATTTTTCGCGAG	1	25 nmol
A-73[43]74[56]	TTTTCGAGCCGGAAGCATAAAGGAAAGGACCTTGTAAAGCC	1	25 nmol
A-74[85]73[92]	TTTTTAGAACCCTTCTCGTAAGAATACGTGGCACATTTT	1	25 nmol

A-75[36]76[36]	TTTTTGAGCTAACGTGCCAGCTTTTT	1	25 nmol
A-75[49]74[36]	TCACATTTGGGGTGCCTAATGAGTTTT	1	25 nmol
A-75[56]38[47]	AATTGCGATAGTAGTAGCATTTT	1	25 nmol
A-76[62]40[47]	TCGGGAACAACGCGTCGGTTGTACCATTTT	1	25 nmol
A-76[85]75[85]	TTTTCTGGCCACCCGCCACTGACAGAGATTTT	1	25 nmol
A-77[36]76[49]	TTTTGCATTAATGAATCGGCACCTGTC	1	25 nmol
A-78[62]42[47]	GGCGCCAAACAGCTTATATTTTAAATTTTT	1	25 nmol
A-78[85]77[85]	TTTTATAAAAGGTTTGAGGCGGGACATTTTTT	1	25 nmol
A-79[36]80[36]	TTTTCACCAGTGAAAGCGGTCCTTTT	1	25 nmol
A-79[49]78[36]	GACGGGCGGGTGGTTTTTCTTTTTTTT	1	25 nmol
A-8[142]63[139]	AAGCCAGAATGCCCTTTTAACTGAGGTTTAA	1	25 nmol
A-8[166]29[166]	TTTTAAACAAATACAGATAGCCTTTT	1	25 nmol
A-8[97]64[70]	TGAACGGAAGAGCAAATATCATTTGCACGTTAGAACCTACCATAGTTGGGAAGGGC	1	25 nmol
A-80[65]84[56]	CCTGAGAGAGATTATTTTTTGACGCGCCAGCCTGGTAA	1	25 nmol
A-80[85]79[85]	TTTTTCACACGCCGCCCTTCAACCAGTATTTT	1	25 nmol
A-81[36]80[49]	TTTTACGCTGGCTGAAATGGTTGCAGC	1	25 nmol
A-82[85]42[73]	TTTTCTCATGGAAATAACGAGCATTTT	1	25 nmol
A-83[36]82[36]	TTTTCAGCAGGCAATATTACCTCAATCGTTTTGCCCTTTT	1	25 nmol
A-83[63]83[85]	CATTGCAACAGGAAAAACGTTTT	1	25 nmol
A-84[85]44[70]	TTTTACTCAAACTATCATAAACAGAACAAG	1	25 nmol
A-85[36]84[36]	TTTTCCTGTTTTGATTAGTATATCCAGAACGAAAATTTTT	1	25 nmol
A-85[56]88[40]	ATAACATTGTAGCAAATCCCTAAGAATAGCCCGAGATAGGTTTT	1	25 nmol
A-86[85]46[73]	TTTTCATCACGCAAATGCCGGAGAAGG	1	25 nmol
A-87[36]86[36]	TTTTGTTCCGAAATCGGCAAATACTTCTTGATGGTGTTTT	1	25 nmol
A-87[63]87[85]	TATAATAAAAGAGTCTGTCTTTT	1	25 nmol
A-88[89]88[63]	TTTTATCAGTGAGGCCACCGAGATCAA	1	25 nmol
A-89[40]89[62]	TTTTGTTGAGTGTTGTTCCGCCA	1	25 nmol
A-9[94]9[111]	GACAAGAACAGCCGCCGC	1	25 nmol
A-90[62]90[40]	CGGTACAGTTTGGAACAAGTTTT	1	25 nmol
A-90[89]89[69]	TTTTATTAAAGGGATTTTAGACAGGAAGAATCCT	1	25 nmol
A-91[40]91[62]	TTTTAGTCCACTATTAAAATCAG	1	25 nmol
A-92[100]52[93]	TGGTTGCTTTCGGTCACCGTTA	1	25 nmol
A-92[131]24[112]	TTTTTAATGCGCCGCTACAGCCACACCAAGCCTGACACCGGAGCGCCA	1	25 nmol
A-92[62]92[40]	TTAGAGAACGTGGACTCCATTTT	1	25 nmol
A-92[76]25[85]	ACGTGCTCTAGGGCAATCATAAAACAGGTTCAGAATTTACCCTGACTATTA	1	25 nmol
A-93[40]93[62]	TTTTACGTCAAAGGGCGAAAGGA	1	25 nmol
A-94[131]54[115]	TTTTGGGAGCCCCCGATTTAAAATCGGTTAA	1	25 nmol
A-94[62]94[40]	AGCGAAAAACCGTCTATCATTTT	1	25 nmol
A-94[76]55[86]	AAGGAAGACCCAAAATTGTAATTTTAACCAATAACGCG	1	25 nmol
A-94[90]92[77]	CGAACGTAGTGTAGGACGAGCACGTATA	1	25 nmol
A-95[108]92[101]	CACTGAGCTTGGTAACCAGGCGCGTACTA	1	25 nmol
A-95[40]54[40]	TTTTGGCGATGGCCCATTAAAATTCGCATTTT	1	25 nmol
A-95[66]50[74]	CATCGGAAGAAGCGGGCGTTCCTCGAGCGGGAGCTAAACTCA	1	25 nmol
A-95[84]54[93]	TTTTGGGGTCGAGGCGTGT	1	25 nmol
A-5[231]3[253]	TCAAGAGCCAGGCGACCCATGTACCGTAACACTTTTT	1	25 nmol
B-0[260]5[257]	TTTTCGAATAATAATTAGGATTAGCTTTT	2	25 nmol
C-anchor-0[260]5[257]	CAACTAAGCCTTTTCGAATAATAATTAGGATTAGCTTTT	3	25 nmol
G-9[64]10[64]	TTTTGGCTGACCTAACAAAGCTTTTT	4	25 nmol
G-17[68]18[68]	TTTTCTAATGCAGATACTCGTTTACCAGTTTT	4	25 nmol
G-18[94]2[78]	CTATACGTAATGAAGTTTCCATTAAACTTTT	4	25 nmol
G-20[97]4[82]	CAGAGGGTCACCAGATTTGTATCATCTTTT	4	25 nmol
G-21[61]22[61]	TTTTTAGCGTCCAATACTGAGCCAATTAGCGGAATCGTCATATTTT	4	25 nmol
G-23[61]6[61]	TTTTAATATTCATTGAGCGCAGACGGTCTTTT	4	25 nmol
G-36[80]12[64]	GCAATCATTGTTGAGATGGTTTAATTTTTTTT	4	25 nmol
G-38[80]14[68]	GTGGAGGCAAAGACGTTGGGAAGTTTT	4	25 nmol
G-40[80]16[68]	TCAGTGCGGGATACCACATTCAATTTT	4	25 nmol
G-5[82]0[85]	TTTTGCCTGATAAATTCCGATATATTTTT	4	25 nmol
G-7[61]8[64]	TTTTAATCATAAGGGAGCATAGGCTTTTT	4	25 nmol
G-89[70]20[61]	GAGAAGTTATTCAAGTAGCTAGTTTAGACTGGATTTT	4	25 nmol
H-9[64]10[48]-FL	TTTTGGCTGACCTAACAAAGCTACCACCTCCTTACTACTCCC	5	25 nmol
H-17[72]18[52]-FL	TTTTCTAATGCAGATACTCGTTTACCAGACCACCTCCTTACTACTCCC	5	25 nmol
H-18[94]2[62]-FL	CTATACGTAATGAAGTTTCCATTAAACACCACCTCCTTACTACTCCC	5	25 nmol
H-20[97]4[66]-FL	CAGAGGGTCACCAGATTTGTATCATCACCACCTCCTTACTACTCCC	5	25 nmol
H-21[61]22[45]-FL	TTTTTAGCGTCCAATACTGAGCCAATTAGCGGAATCGTCATAACCACCTCCTTACTACTCCC	5	25 nmol
H-23[61]6[45]-FL	TTTTAATATTCATTGAGCGCAGACGGTCACCACCTCCTTACTACTCCC	5	25 nmol
H-36[80]12[48]-FL	GCAATCATTGTTGAGATGGTTTAATTTACCACCTCCTTACTACTCCC	5	25 nmol
H-38[80]14[52]-FL	GTGGAGGCAAAGACGTTGGGAAGACCACCTCCTTACTACTCCC	5	25 nmol
H-40[80]16[52]-FL	TCAGTGCGGGATACCACATTCAAACCACCTCCTTACTACTCCC	5	25 nmol
H-5[82]0[69]-FL	TTTTGCCTGATAAATTCCGATATACCACCTCCTTACTACTCCC	5	25 nmol
H-7[61]8[48]-FL	TTTTAATCATAAGGGAGCATAGGCTACCACCTCCTTACTACTCCC	5	25 nmol
H-89[70]20[45]-FL	GAGAAGTTATTCAAGTAGCTAGTTTAGACTGGAACCACCTCCTTACTACTCCC	5	25 nmol
I-B-4[257]0[237]	TTTTGGGGTTTTGCTCAGTAAAGGATTTTTTCACG	6	25 nmol
I-B-0[236]4[223]	TTGAAAATAGAAACAGTACAAACTACAAATAGGAGATAAGTG	6	25 nmol
I-B-2[253]1[260]	TTTTGAGTTTCGTCACGGAACAACTAAAGGAATTGTTTT	6	25 nmol
J-3'-0[244]1[237]	TTTTCACGTTGAAAATAGAAA	7	25 nmol
J-3'-2[253]4[223]	TTTTGAGTTTCGTCACCAGTACAAACTACAAATAGGAGATAAGTG	7	25 nmol
J-3'-4[257]5[244]	TTTTGGGGTTTTGCTCAGTAAAGGATT	7	25 nmol
K-3’add	GGAACAACTAAAGGAATTGCCGGCCGGCGGC	8	25 nmol
**Functionalized Oligonucleotides**			
cy5 label	/5Cy5/GGGAGTAGTAAGGAGGTGGT	N/A	100 nmol, HPLC
texas red label	/5TexRd-XN/GGGAGTAGTAAGGAGGTGGT	N/A	100 nmol, HPLC
**Aptamer Template Oligonucleotides**			
apt for SPOT, anti-sense strand	CCGGCCGGCGGCGGGAGAAGCTTCTGTGGTGGCCCTCTTTTAAGGACTTTCCCCACCCAATTGCGCCACGACGTTTC GTGCTCCCATCGCAGTCCAGAAGCTTCTCCCTATAGTGAGTCGTATTAG	N/A	4 nmol Ultramer
apt for SPOT, sense strand	CTAATACGACTCACTATAGGGAGAAGCTTCTGGACTGCGATGGGAGCACGAAACGTCGTGGCGCAATTGGGTGGGGA AAGTCCTTAAAAGAGGGCCACCACAGAAGCTTCTCCCGCCGCCGGCCGG	N/A	4 nmol Ultramer
free apt, anti-sense strand	CTAATACGACTCACTATAGGGAGCTTCTGGACTGCGATGGGAGCACGAAACGTCGTGGCGCAATTGGGTGGGGAAAG TCCTTAAAAGAGGGCCACCACAGAAGCTCCC	N/A	4 nmol Ultramer
free apt, sense strand	GGGAGCTTCTGTGGTGGCCCTCTTTTAAGGACTTTCCCCACCCAATTGCGCCACGACGTTTCGTGCTCCCATCGCAGT CCAGAAGCTCCCTATAGTGAGTCGTATTAG	N/A	4 nmol Ultramer
rigid apt for SPOT, anti-sense strand	CAACTAAGCCAGCTTCTGTGGTGGCCCTCTTTTAAGGACTTTCCCCACCCAATTGCGCCACGACGTTTCGTGCTCCCA TCGCAGTCCAGAAGCTCAATTCCTTTAGTTGTTCCTATAGTGAGTCGTATTAG	N/A	4 nmol Ultramer
rigid apt, sense strand	CTAATACGACTCACTATAGGAACAACTAAA	N/A	25 nmol

Table S2 – Mixtures of staples required to produce SPOTs and their derivatives or precursors, related to STAR Methods.

Construct	Required Staple Mixes from Table S1	Required Additional Reagents
Signpost origami (SPO)	1, 2, 4, 6	-
SPO with Texas red label	1, 2, 5, 6	Texas red labeled oligo
SPO with cy5 label	1, 2, 5, 6	cy5 labeled oligo
SPOT (SPO with aptamer)	1, 2, 4, 7, 8	Transcribed apatmer
SPOT with Texas red label	1, 2, 5, 7, 8	aptamer, Texas red label
SPOT with cy5 label	1, 2, 5, 7, 8	aptamer, cy5 label
SPOT with rigid aptamer attachment	1, 3, 4, 8	rigid aptamer

Table S3 – Primers used for cloning of fluorescent protein genes, related to STAR Methods.

Description	Primer type	Sequence
Primers for insertion of fluorescent protein genes in pEP98-gB	forward	GGCGGGAATTCATGGTGAGCAAGGGCGAG
	reverse	GGCGGCTCGAGCTTGTACAGCTCGTCCATGC
Primers for insertion of TEV restriction site as linker into mCherry-gB in pEP98	forward	GGCGGGAATTCCGCCGACGCCACCAGG
	reverse	gBGGCGGCTCGAGAACCTGTACTTCCAGTCCGCTCCGACTTCCCCCGG
Primers for ligation independent cloning of fluorescent proteins into pET-28, plasmid	forward	CACCACCACCACTGA
	reverse	AGTGGTGGTGGTGCTTACTTGTACAGCTC
Primers for ligation independent cloning of fluorescent proteins into pET-28, insert	forward	AGATTGGTGAGAATTTATATTTTCAGGGCCATATGGTGAGCAAGG
	reverse	AGTGGTGGTGGTGCTTACTTGTACAGCTC
